# Versatile synthesis of amino acid functionalized nucleosides via a domino carboxamidation reaction

**DOI:** 10.3762/bjoc.10.268

**Published:** 2014-11-04

**Authors:** Vicky Gheerardijn, Jos Van den Begin, Annemieke Madder

**Affiliations:** 1 Department of Organic and Macromolecular Chemistry, Organic and Biomimetic Chemistry Research Group, Ghent University, Krijgslaan 281 S4, 9000 Ghent, Belgium

**Keywords:** amino acids, bioorganic chemistry, carboxamidation reaction, chemically modified, DNA nucleosides, nucleic acids

## Abstract

Functionalized oligonucleotides have recently gained increased attention for incorporation in modified nucleic acid structures both for the design of aptamers with enhanced binding properties as well as the construction of catalytic DNA and RNA. As a shortcut alternative to the incorporation of multiple modified residues, each bearing one extra functional group, we present here a straightforward method for direct linking of functionalized amino acids to the nucleoside base, thus equipping the nucleoside with two extra functionalities at once. As a proof of principle, we have introduced three amino acids with functional groups frequently used as key-intermediates in DNA- and RNAzymes via an efficient and straightforward domino carboxamidation reaction.

## Introduction

For decades DNA has been known as the carrier of the genetic information. Only recently, the use of synthetic oligonucleotides and their modified analogues for a range of therapeutic and diagnostic purposes [1], including antisense therapy [2–3], antigene therapy [4–5] and SNP (Single Nucleotide Polymorphism) detection [6] has gained major interest. Due to their predictable and well-investigated structure, the strength of nucleic acids for a series of applications such as DNA and RNA based drugs [7], drug delivery systems [8–9], DNA-biosensors [10–11] and potential catalysts has now firmly been recognized. Rather than using unmodified oligonucleotides, providing additional functional groups can lead to even higher activities and selectivities. Indeed, people have realized that equipping the nucleic acid scaffold with protein side chain-like moieties may assist in the design of a broad scope of functionalized oligonucleotides with various characteristics. This can be enabled by techniques such as solid phase synthesis, post synthetic modifications or enzymatic incorporation of modified analogues.

Originating from research into aptamers as strong and selective binders [12–14], several research groups are investigating the creation and synthesis of new DNA or RNA catalysts, also called DNAzymes [15–17] and RNAzymes [10,16,18–21], respectively. Most catalytic nucleic acids are generated using the SELEX (Systematic Evolution of Ligands by EXponential enrichment) protocol [22–25] that generates a library of potential catalysts, which are then screened for catalytic potential using natural substrates or transition state analogues. Although examples [26–29] exist illustrating the introduction of modified residues and by that the development of new catalysts*,* the SELEX protocol becomes more labor intensive and reproducibility of results is often a problem. Moreover achieving control in terms of number, positioning and exact location of the desired catalytic moieties is far from straightforward. In contrast to the existing variety of rather complicated and unpredictable RNA based ribozyme-like structures, oligonucleotide duplexes have a stable and predictable structure allowing the design of engineered active sites through the carefully planned introduction of extra catalytic functionalities via solid phase DNA synthesis. We have further recently shown that the combination of solid phase synthesis and molecular modeling combined with advanced NMR techniques offers the possibility to predict and control the positioning of catalytic functions within a DNA duplex [30].

**Figure 1:**
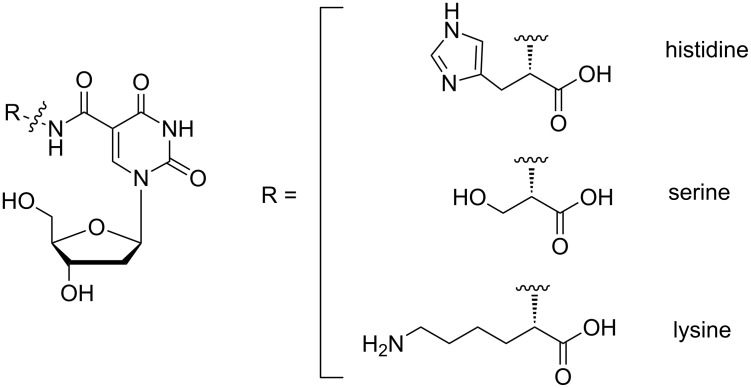
Amino acid functionalized nucleosides.

Chemical modification of nucleic acids can be performed on different positions, including the backbone, the sugar unit and the heterocyclic base, whereby base modification is the most common as it causes only minor disturbance in the helical structure [31]. Depending on the position of the incorporation on the nucleoside structure, the introduced functionalities can be pointed towards the major or the minor groove of the duplex. Modification of position 1’ and 2’ will orient substituents towards the minor groove while positions 5, 7 and 8 on the base allow directing incorporated substituents in the major groove. One can further imagine that introduction of extra functionalities can have an impact on the duplex stability and final structure of the double helix. This was illustrated in previous research in our laboratory where introduction of modified nucleosides on the 2’-position in a DNA double helix resulted in a destabilization of the duplex of 5 °C per modified unit [32]. Although this destabilization depends on many different aspects, such as the type and length of the linker and the position of the modification, it is important to minimize destabilization as much as possible, more specifically when introduction of more than one modification is desired. To date most research groups have focused on the introduction of only one extra functionality per nucleoside, mainly for synthetic reasons, and preferably making use of commercially available building blocks. We therefore considered the incorporation of moieties containing multiple functionalities on a nucleoside residue and present a straightforward method for the introduction of side chain functionalized amino acids onto nucleoside building blocks (Figure 1). Following the event of solid phase peptide synthesis, a large range of amino acids with different protection schemes and stereochemistry is currently commercially available. We here illustrate a methodology for direct incorporation of amino acids via a short synthetic pathway offering the added benefit of introducing multiple functional groups at the same time.

Indeed, whereas the α-amino group is used to ensure linkage to the nucleoside building block, both the side chain functional group and the α-carboxylate end up as extra functionalities on the nucleoside. As proof of principle and with the catalytic serine–histidine–aspartate catalytic triad of serine proteases in mind, we illustrate the preparation of three different nucleosides equipped with three functional amino acids containing hydroxy, imidazole, amine and carboxylate groups for later incorporation into nucleic acid duplexes via solid phase DNA synthesis [33].

## Results and Discussion

As mentioned earlier, a number of research groups have used the SELEX or in vitro selection protocol for the incorporation of amino acid-like modifications into DNA or RNA where there is no need for protection of the functional groups. For example, many imidazole and amine functionalities were incorporated to increase the structural diversity and catalytic potential of nucleic acids [34–37]. Most of these functionalities are introduced into the triphosphate building blocks using palladium-based coupling chemistry to the pyrimidine C5 or the 7-position of 7-deaza-2’-deoxyadenosine [38–40]. During enzymatic incorporation the extra functionalities do not need to be protected. However, solid phase DNA synthesis implies an appropriate protection of the extra functionalities on the corresponding nucleoside building blocks to avoid side-reactions during DNA synthesis. Not only should these protecting groups be stable under all chemical conditions used in the synthesis pathway, they should also be stable under the DNA synthesis conditions and easily removable after assembly of the desired chain without DNA damage.

In the current study, we have chosen to couple three amino acids, which contain functional groups commonly used in SELEX approaches to modify DNA or RNA, to 5-iodo-2’-deoxyuridine. We describe the direct and linker-less introduction of histidine, serine and lysine derivatives onto nucleosides via a straightforward and easy domino carboxamidation reaction. Previously the groups of Gait and Eaton [41–43] have used this reaction to couple histamine or simple amine derivatives to both 5-iodo-2’-deoxyuridine and purine nucleosides. Although a large number of imidazole modified pyrimidine and purine derivatives for solid phase synthesis have been described to date [41,44–47], we believe that the reactions described here serve as an ideal model system, which can be extended to other commercially available amino acid derivatives and nucleosides.

**Scheme 1:**
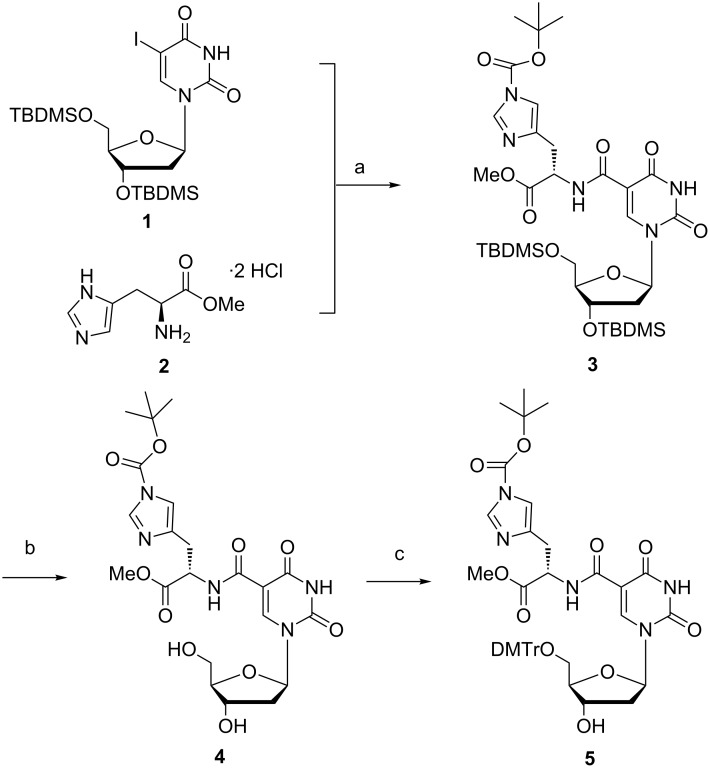
Reagents and conditions: a) i. Et_3_N, Pd(PPh_3_)_4_, THF, CO (4 bar), 70 °C, 48 h, ii. Et_3_N, di-*tert*-butyl dicarbonate, DMF, rt, 15 min (79%); b) Et_3_N·3HF, Et_3_N, THF, rt, overnight (72%); c) DMTr-Cl, pyridine, 0 °C, 7 h (52%).

For the introduction of histidine onto the nucleoside as shown in Scheme 1, we chose to protect the free 3’ and 5’-hydroxy groups with *tert*-butyldimethylsilyl (TBDMS) groups **1** to avoid side reactions during the next step, the carboxamidation reaction [48]. While protected histidine is commercially available, we have synthesized histidine as a methyl ester (**2**) with thionyl chloride in methanol in good yield according to literature procedures [49]. Although basic hydrolysis of alkyl esters has been shown to imply long reaction times, side-product formation and low reaction yields [50], we found that methyl esters within DNA are hydrolysed under basic conditions even at room temperature using aqueous ammonia provided prolonged reaction times are respected [51]. Inspired by the results of Perrin and Joyce [26,52], who introduced a nucleoside containing an unprotected imidazole functionality into the DNA synthesis cycle without problems, we first tried to leave the imidazole unprotected in order to avoid extra protection and deprotection steps in the reaction sequence. However, in view of problems arising during the separation by column chromatography after the CO insertion reaction with an unprotected histidine methyl ester, we decided to protect the imidazole functionality immediately after the reaction with the *tert*-butyloxycarbonyl (*t-*Boc) protecting group [53], which is compatible with the reagents used during nucleoside- and oligonucleotide synthesis [45,54]. While the *t*-Boc group can be removed with a 10% TFA solution [54], the acid labile DNA can suffer from depurination after treatment with acid. According to literature, the *t-*Boc group can also be cleaved during standard deprotection procedures with saturated ammonia after oligonucleotide synthesis [41,55]. In view of all these considerations, coupling of the TBDMS-protected nucleoside **1** with histidine methyl ester **2** followed by in situ *t-*Boc protection of the free hydrogen on the imidazole functionality gave the desired compound **3** in satisfying yield.

In the next step of the synthesis, the TBDMS-protecting groups needed to be removed. First attempts using *tetra*-butylammonium fluoride (TBAF) resulted in degradation of the starting material (results not shown). Indeed, several reports in literature highlight the strong basic character of the fluoride anion (F^−^) that can cause decomposition of or nucleophilic attack on the nucleoside [56–57]. Manfredini et al. have shown that selective deprotection of the TBDMS groups in the presence of a *t-*Boc protecting group is possible when using Et_3_N·3HF [58] and also in our case using Et_3_N·3HF in THF resulted in product **4** with good purity and yield (72%) [59]. Dimethoxytritylation could be accomplished under standard conditions and the corresponding derivative **5** was isolated and purified without problems.

**Scheme 2:**
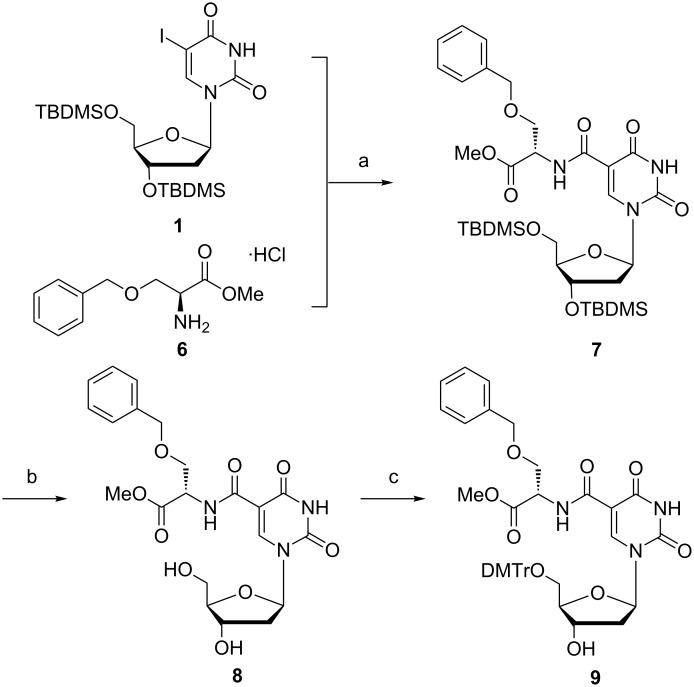
Reagents and conditions: a) Et_3_N, Pd(PPh_3_)_4_, THF, CO (4 bar), 48 h, 70 °C (68%); b) Et_3_N·3HF, Et_3_N, THF, overnight, rt (86%); c) DMTr-Cl, pyridine, DCM, overnight, rt (82%).

As a second example we decided to employ serine as amino acid for introduction onto the nucleoside. In view of the earlier illustrated need for imidazole protection during introduction of histidine (vide supra), we used commercially available benzyl protected serine **6** to avoid problems due to the free hydroxy group of the serine during the carboxamidation reaction (Scheme 2). After DNA synthesis, the benzyl group can be cleaved using hydrogen and palladium on carbon. To avoid overreduction of the pyrimidine double bond, transfer hydrogenation with cyclohexene as hydrogen source and 10% palladium on carbon should be used [60–62]. As the methyl ester derivative of the amino acid is commercially available but rather expensive, we performed the esterification reaction on the benzyl protected serine to deliver product **6** in good yield and purity after recrystallization in cold diethyl ether (64%) [63]. Coupling of protected serine methyl ester **6** with TBDMS-protected nucleoside **1** in the presence of tetrakis(triphenylphosphine)palladium(0) and Et_3_N in THF under CO atmosphere (4 bar) gave the desired product **7**. After deprotection of the TBDMS groups to yield **8** under similar reaction conditions as used before to obtain product **4**, the free 5’-hydroxy group was selectively protected with DMTr-Cl to obtain the modified desired nucleoside **9** in good yield and purity.

**Scheme 3:**
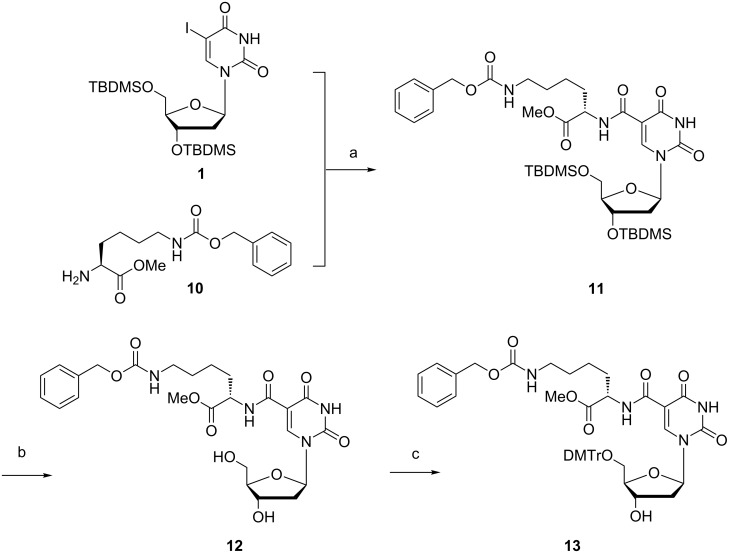
Reagents and conditions: a) Et_3_N, Pd(PPh_3_)_4_, THF, CO (4 bar), 48 h, 70 °C (90%); b) Et_3_N·3HF, Et_3_N, THF, overnight, rt (43%); c) DMTr-Cl, pyridine, DCM, overnight, rt (9%).

As the functional groups of histidine (imidazole) and lysine (cationic amine) are known to be present in numerous enzyme active sites and capable of general acid–general base catalysis, we have finally chosen to modify a nucleoside with a lysine amino acid derivative as a third example of our current strategy, as shown in Scheme 3. Protected lysine **10** is commercially available and was coupled with the TBDMS-protected nucleoside **1** to afford product **11** in high yield. After deprotection of the *tert*-butyldimethylsilyl protecting groups using the above described protocol, modified nucleoside **12** could be selectively protected at the free 5’-hydroxy group with DMTr-Cl to afford **13**. Compared to the previous reaction schemes, this final reaction suffers more from the steric hindrance as compared to the other amino acids that show less bulky substituents.

## Conclusion

We here illustrated the efficient application of a simple carboxamidation procedure for the synthesis of modified nucleosides featuring two extra functional groups. Because hydrolysis of the methyl ester and removal of the *t-*Boc is performed during the standard cleavage of solid support using NH_4_OH and benzyl and benzoyl protecting groups can be removed by hydrogenation [64], only one extra deprotection step is needed after incorporation. After transformation of the modified nucleosides into the corresponding phosphoramidite building blocks **5**, **9** and **13** are amenable to incorporation in nucleic acids through standard DNA synthesis methodology. The described protocol relies on commercially available amino acid derivatives and should be applicable to a large series of natural and unnatural amino acids.

## Supporting Information

File 1 Experimental procedures, characterization data, and ^1^H and ^13^C NMR spectra of new compounds.
