# Supplementary material for: Versatile synthesis of amino acid functionalized nucleosides via a domino carboxamidation reaction
Source: Beilstein J Org Chem. 2014 Nov 4;10:2566–72. doi: 10.3762/bjoc.10.268 (PMC4222392; doi:10.3762/bjoc.10.268)

Supporting Information

for

Versatile synthesis of amino acid functionalized nucleosides via a domino carboxamidation reaction

Vicky Gheerardijn, Jos Van den Begin and Annemieke Madder*

Address: Department of Organic and Macromolecular Chemistry, Organic and Biomimetic Chemistry Research Group, Ghent University, Krijgslaan 281 S4, 9000 Ghent, Belgium

Email: Annemieke Madder* - Annemieke.Madder@UGent.be

* Corresponding author

Experimental procedures, characterization data, and ^{1}H and ^{13}C NMR spectra of new compounds

Tabel of Contents

Experimental procedures of product **3–5**, **7–9**, **11–13**..**S2**

^{1}H NMR and ^{13}C NMR of product **3**..**S9**

^{1}H NMR and ^{13}C NMR of product **4**..**S11**

^{1}H NMR and ^{13}C NMR of product **5**..**S13**

^{1}H NMR and ^{13}C NMR of product **7**..**S15**

^{1}H NMR and ^{13}C NMR of product **8**..**S17**

^{1}H NMR and ^{13}C NMR of product **9**..**S19**

^{1}H NMR and ^{13}C NMR of product **11**..**S21**

^{1}H NMR and ^{13}C NMR of product **12**..**S23**

^{1}H NMR and ^{13}C NMR of product **13**..**S25**

Experimental Section

General Remarks: All chemicals and solvents (Sigma-Aldrich, Fluka, Acros, Novabiochem, Iris Biotech GmbH) were purchased and used without any further purification, except dichloromethane, which was distilled from CaH_2 prior to use. All reactions were performed under argon or nitrogen with magnetic stirring and were monitored by thin-layer chromatography (TLC) using SIL G-25 UV254 pre-coated silica gel plates (0.25 mm thickness). Reactions under CO atmosphere were carried out in a Parr-High-Pressure reaction vessel with particular care. The ultra sonication was performed using a sonic bath (42 kHz, Bransonic, 2510E-MT, USA) at ambient temperature. TLC plates were visualized by using anisaldehyde (5% anisaldehyde in ethanol with 1% sulfuric acid) or PMA (5% phosphomolybdic acid in ethanol) solutions. Flash column chromatography was performed using BIOSOLVE silica gel (0.063–0.200 mm particle size, 20–30 g silica/1 g compound/ KieselgelMerck, 230–400 mesh, Type 9385, 60 Angström). ^{1}H NMR spectra were recorded at 300 MHz, ^{13}C NMR spectra were recorded at 75 MHz. Chemical shifts (δ) are reported in units of parts per million (ppm), with the residual ^{1}H or ^{13}C peaks of the solvent used as internal standards ($CDCl_3$: δH = 7.26 ppm and δC = 77.16 ppm, CD_3OD: δH = 3.31 ppm and δC = 49.00 ppm). The following abbreviations are used to explain the observed multiplicities: s, singlet; d, doublet; t, triplet; q, quadruplet; m, multiplet; br, broad; band, several overlapping signals; AB, AB system with strongly skewed signals; app, indicates an "apparent" multiplicity, for which only the observed average coupling constant can be quoted, in absence of information on the real *J* values. Where given, assignments of resonances were confirmed by standard COSY and HSQC 2D NMR experiments. High resolution mass spectra (HRMS) were recorded with an Agilent Accurate-Mass Quadrupole Time-of-Flight mass spectrometer.

Synthesis of the histidine modified nucleoside (3): The TBDMS-protected nucleoside **1** (500 mg, 0.86 mmol) was suspended in THF (20 mL) in a Parr-High-Pressure reaction vessel and the protected histidine **2** (622 mg, 2.6 mmol), Et_3N (1.2 mL, 8.6 mmol) and $Pd(PPh_3)_4$ (99.4 mg, 0.09 mmol) were added in the order listed. The reaction mixture was incubated at 70 °C under 50 psi (~4 bar) carbon monoxide for 48 h. The reaction mixture was cooled down to room temperature, filtered over celite and concentrated under vacuum. The residue was taken up in anhydrous DMF (20 mL) and to the stirring solution under argon, Et_3N (350

μL, 2.6 mmol) and *tert*-butyl dicarbonate (282 mg, 1.3 mmol) was added. After 15 min, the reaction was quenched with anhydrous methanol (1 mL) and again concentrated to an oil. The oil was purified using flash chromatography on silica gel with 3 % MeOH in DCM to yield product **3** as a yellow oil (511 mg, 0.68 mmol, 79%). R_f (DCM: MeOH 9:1) = 0.63; ^{1}H NMR (300 MHz, $CDCl_3$, 25°C): δ 9.21 (d, *J* = 7.5 Hz, 1H, -N*H*), 8.65 (s, 1H, *H6*), 8.33 (s br, 1H, pyrimidine-N*H*), 8.00 (app d, *J* = 1.1 Hz, 1H, H*10*), 7.17 (s br, 1H, H*9*), 6.20 (dd, *J* = 5.7 and 7.8 Hz, 1H, *H1*'), 4.98 (app dt, *J* = 5.8 and 7.5 Hz, 1H, *H7*), 4.40 (dt, *J* = 2.0 and 5.9 Hz, 1H, *H3*'), 4.03 (td, *J* = 2.0 and 3.7 Hz, 1H, *H4*'), 3.77 (m, 2H, *H5'/H5*''), 3.73 (s, 3H, -OCH_3), 3.16 (d, *J* = 5.8 Hz, 2H, *H8'/H8*''), 2.39 (ddd AB, *J* = 2.0, 5.7 and 13.3 Hz, 1H, *H2*'/*H2*''), 2.06 (ddd AB, *J* = 5.8, 7.8 and 13.3 Hz, 1H, *H2*'/*H2*''), 0.89 (s, 9H, -*tBu*), 0.87 (s, 9H, -*tBu*), 0.09 (s, 6H, -CH_3), 0.07 (s, 6H, -CH_3); ^{13}C NMR (75 MHz, $CDCl_3$, 25°C): δ 171.7 (C), 162.7 (C), 161.6 (C), 149.7 (C), 147.0 (C), 146.3 (CH), 138.8 (C), 132.2 (CH), 128.5 (CH), 114.8 (CH), 106.0 (C), 88.8 (CH), 87.0 (CH), 85.5 (C), 73.1 (CH_2), 63.4 (CH_2), 52.5 (CH), 52.4 (CH_3), 41.6 (CH_2), 30.4 (CH_2), 28.0 (CH_3), 26.0 (CH_3), 25.8 (CH_3), 18.5 (C), 18.1 (C), -4.6 (CH_3), -4.7 (CH_3), -5.4 (CH_3), -5.5 (CH_3); HRMS (ESI): *m/z*: calcd for $C_{34}H_{56}O_{10}N_5Si_2$: 750.3565 $[M-H]^-$; found: 750.3578.

General synthesis for the deprotection of TBDMS-protected nucleosides 3, 7 and 11 to afford the modified compounds 4, 8 and 12: The protected nucleoside (0.4–0.6 mmol) was dissolved in dry THF (3–5 mL) and $Et_3N{\cdot}3HF$ with an additional amount of dry Et_3N were added. The reaction mixture was stirred overnight at room temperature under argon atmosphere. The solvent was removed under reduced pressure and the resulting crude was purified with flash chromatography on silicagel using a gradient 2–5% MeOH in DCM to yield a white foam (43–86%).

Experimental details of product (4): Reaction of product **3** (409 mg, 0.54 mmol) and $Et_3N{\cdot}3HF$ (0.53 mL, 3.3 mmol) with an additional amount of dry Et_3N (0.45 mL, 3.3 mmol) according to the general procedure described above, gave the deprotected nucleoside **4** (204 mg, 72%). R_f (DCM: MeOH 9:1) = 0.37; ^{1}H NMR (300 MHz, $CDCl_3$, 25°C): δ 9.94 (d, *J* = 7.9 Hz, 1H, -N*H*), 8.83 (s, 1H, *H6*), 8.08 (app d, *J* = 1.1 Hz, 1H, H*10*), 7.16 (s br, 1H, H*9*), 6.13 (t, *J* = 5.2 Hz, 1H, *H1*'), 5.09 (app dt, *J* = 5.0 and 7.9 Hz, 1H, *H7*), 4.56 (q, *J* = 5.2 Hz, 1H, *H3*'), 4.07 (q, *J* = 5.2 Hz, 1H, *H4*'), 3.89 (m, 2H, *H5'/H5*''), 3.71 (s, 3H, -OCH_3), 3.29 (dd AB, *J* = 5.0 and 14.7 Hz, 1H, *H8'/H8*''), 3.15 (m, 1H, *H8'/H8*''), 2.44 (q, *J* = 5.2 Hz, 2H, *H2*'/*H2*''), 0.89 (s, 9H, -*tBu*); ^{13}C NMR (75 MHz, $CDCl_3$, 25°C): δ 171.5 (C), 162.9 (C),

162.2 (C), 149.7 (C), 148.4 (CH), 146.9 (C), 138.2 (C), 115.0 (CH), 105.2 (C), 88.7 (CH), 88.3 (CH), 86.0 (C), 70.7 (CH), 61.9 (CH_2), 52.6 (CH/CH_3), 52.5 (CH_3), 40.4 (CH_2), 30.2 (CH_2), 28.0 (CH_3); HRMS (ESI): *m/z*: calcd for $C_{22}H_{28}O_{10}N_5$: 522.1836 $[M-H]^-$; found: 522.1856.

General synthesis of the modified dimethoxytrityl analogues 5, 9 and 13 from the corresponding modified nucleosides 4, 8 and 12: The starting nucleoside (0.16–0.20 mmol) was co-evaporated three times with pyridine and dried overnight under vacuum. The modified nucleoside was dissolved in dry pyridine (1–4 mL) and dry DCM (0.5–2 mL). 4,4'-dimethoxytrityl chloride, dissolved in dry pyridine (1–2 mL) and dry DCM (0.5–1 mL), was added dropwise to this solution at 0 °C. The reaction was stirred overnight at room temperature under argon atmosphere for 7 h. The reaction was quenched with anhydrous MeOH (1–2 mL) at 0 °C and the solvent was removed under reduced pressure. The remaining residue (yellow oil) was dissolved in DCM (5 mL). The organic phase was washed with 2x sat. sol. $NaHCO_3$ (10 mL) and 1x with brine (10 mL). The collected organic phases were dried on Na_2SO_4 and the solvent was evaporated in vacuo. The remaining residue was purified by flash chromatography on silica gel using a gradient of 0-5 % methanol in DCM + 1% Et_3N to give the desired product (9–82%).

Experimental details of product (5): Reaction of product **4** (102 mg, 0.20 mmol) with DMTr-Cl (135 mg, 0.40 mmol) in pyridine 4 mL and DCM 2 mL according to the general procedure described above, gave the protected nucleoside **5** (84 mg, 52%). R_f (DCM: MeOH 9:1) = 0.43; ^{1}H NMR (300 MHz, $CDCl_3$, 25°C): δ 9.29 (d, *J* = 7.4 Hz, 1H, -N*H*), 8.53 (s, 1H, *H6*), 8.01 (app d, *J* = 1.1 Hz, 1H, H*10*), 7.41-7.19 (band, 10H, -DMTr*H* + *H9*), 8.85 (d, *J* = 8.9 Hz, 4H, -DMTr*H*), 6.15 (app t, *J* = 6.4 Hz, 1H, *H1*'), 4.93 (app dt, *J* = 5.7 and 7.4 Hz, 1H, *H7*), 4.33 (dt, *J* = 5.3 and 6.4 Hz, 1H, *H3*'), 3.95 (app q, *J* = 5.3 Hz, 1H, *H4*'), 3.78 (s, 6H, -OCH_3), 3.69 (s, 3H, -OCH_3), 3.48 (dd AB, *J* = 5.3 and 10.2 Hz, 1H, *H5*'/*H5*''), 3.34 (dd AB, *J* = 5.3 and 10.2 Hz, 1H, *H5*'/*H5*''), 3.21 (dd AB, *J* = 5.7 and 14.9 Hz, 1H, *H8*'/*H8*''), 3.12 (dd AB, *J* = 5.7 and 14.9 Hz, 1H, *H5*'/*H5*''), 2.45 (ddd AB, *J* = 5.3, 6.4 and 13.7 Hz, 1H, *H2*'/*H2*''), 2.20 (dt AB, *J* = 6.4 and 13.7 Hz, 1H, *H2*'/*H2*''), 1.58 (s, 9H, -*tBu*); ^{13}C NMR (75 MHz, $CDCl_3$, 25°C): δ 171.7 (C), 162.4 (C), 161.5 (C), 158.7 (C), 149.4 (C), 147.0 (C), 146.3 (CH), 144.7 (C), 138.7 (C), 135.7 (C), 130.2 (CH), 128.2 (CH), 128.1 (CH), 127.1 (CH), 114.8 (CH), 113.43 (CH), 106.1 (C), 87.0 (C), 86.2 (CH), 85.6 (CH), 72.6 (CH), 63.8 (CH_2),

55.4 (CH/CH_3), 52.5 (CH/CH_3), 46.0 (C), 40.5 (CH_2), 30.4 (CH_2), 28.0 (CH_3); HRMS (ESI): *m/z*: calcd for $C_{43}H_{48}O_{12}N_5$: 826.3299 $[M+H]^+$; found: 826.3279.

General synthesis of 5-carboxamide modified analogues 7 and 11 from the corresponding protected TBDMS-protected compound 1: The protected nucleoside **1** (0.9–1.2 mmol) was suspended in THF (25 mL) in a Parr-High-Pressure reaction vessel and the protected amino acid **6** and **10**, Et_3N and $Pd(PPh_3)_4$ were added in the order listed. The reaction mixture was incubated at 70 °C under 50 psi (~4 bar) carbon monoxide for 48 h. The reaction mixture was cooled down to room temperature, filtered over celite and concentrated under vacuum. The remaining yellow oil was then purified on a silica gel column using 0–2% methanol in DCM as eluent to give the modified analogue (68–90%).

Synthesis of the serine modified nucleoside (7): Reaction of 5-iodo-3',5'-(*O*-di-*tert*-butyldimethylsilyl)-2'-deoxyuridine (**1**, 528 mg, 0.91 mmol) and *O*-benzyl-*L*-serine methyl ester **6** (634 mg, 2.6 mmol) according to the general procedure described above, gave the deprotected nucleoside **7** as a white solid (430 mg, 68%). R_f (DCM: MeOH 9:1) = 0.64; ^1H NMR (300 MHz, $CDCl_3$, 25°C): δ 9.31 (d, *J* = 7.7 Hz, 1H, -N*H*), 9.22 (s br, 1H, pyrimidine-N*H*), 8.61 (s, 1H, *H6*), 7.30-7.18 (band, 5H, -Ar*H*), 6.17 (dd, *J* = 5.8 and 7.5 Hz, 1H, *H1*'), 4.83 (app dt, *J* = 3.7 and 7.7 Hz, 1H, *H7*), 4.54 (d AB, *J* = 12.4 Hz, 1H, *H9*'/*H9*''), 4.48 (d AB, *J* = 12.4 Hz, 1H, *H9*'/*H9*''), 4.37 (dt, *J* = 2.4 and 6.0 Hz, 1H, *H3*'), 4.00 (td, *J* = 2.4 and 4.1 Hz, 1H, *H4*'), 3.89 (dd AB, *J* = 3.7 and 9.7 Hz, 1H, *H8*'/*H8*''), 3.74 (d, *J* = 4.1 Hz, 2H, *H5*'/*H5*''), 3.70 (s, 3H, -OCH_3), 3.66 (dd AB, *J* = 3.7 and 9.7 Hz, 1H, *H8*'/*H8*''), 2.37 (ddd AB, *J* = 2.4, 5.8 and 13.5 Hz, 1H, *H2*'/*H2*''), 2.03 (ddd AB, *J* = 5.8, 6.0 and 13.5 Hz, 1H, *H2*'/*H2*''), 0.86 (s, 9H, -*tBu*), 0.82 (s, 9H, -*tBu*), 0.05 (s, 3H, -CH_3), 0.04 (s br, 6H, -CH_3), 0.03 (s, 3H, -CH_3); ^{13}C NMR (75 MHz, $CDCl_3$, 25°C): δ 170.6 (C), 162.6 (C), 161.6 (C), 149.5 (C), 146.5 (CH), 137.6 (C), 128.4 (CH), 127.8 (CH), 127.6 (CH), 105.9 (C), 88.9 (CH), 87.2 (CH), 73.3 (CH_2), 73.0 (CH), 69.6 (CH_2), 53.0 (CH), 52.6 (CH_3), 52.5 (CH), 41.7 (CH_2), 26.0 (CH_3), 25.9 (CH_3), 18.5 (C), 18.1 (C), -4.6 (CH_3), -4.8 (CH_3), -5.4 (CH_3), -5.5 (CH_3); HRMS (ESI): *m/z*: calcd for $C_{33}H_{54}O_9N_3Si_2$: 692.3398 $[M+H]^+$; found: 692.3390.

Experimental details of the deprotected product (8): Reaction of the modified nucleoside **7** (353 mg, 0.51 mmol) and $Et_3N{\cdot}3HF$ (0.50 mL, 3.1 mmol) with an additional amount of dry Et_3N (0.40 mL, 3.0 mmol) according to the general procedure described above, gave the deprotected nucleoside **8** as a white foam (204 mg, 86%). R_f (DCM: MeOH 9:1) = 0.29; ^1H

NMR (300 MHz, CD_3OD, 25°C): δ 8.84 (s, 1H, *H6*), 7.28 (m, 5H, -Ar*H*), 6.26 (app t, *J* = 6.3 Hz, 1H, *H1*'), 4.78 (app t, *J* = 3.3 Hz, 1H, *H7*), 4.54 (d, *J* = 1.7 Hz, 2H, *H9*'/*H9*''), 4.40 (dt, *J* = 3.4 and 6.3 Hz, 1H, *H3*'), 3.98 (app q, *J* = 3.4 Hz, 1H, *H4*'), 3.95 (dd, *J* = 3.3 and 9.8 Hz, 1H, *H8*'/*H8*''), 3.75 (dd, *J* = 3.3 and 9.8 Hz, 1H, *H8*'/*H8*''), 3.74 (s, 3H, -OCH_3), 3.83-3.70 (band, 2H, *H5*'/*H5*''), 2.40 (ddd AB, *J* = 3.4, 6.3 and 13.5 Hz, 1H, *H2*'/*H2*''), 2.25 (dt AB, *J* = 6.3 and 13.5 Hz, 1H, *H2*'/*H2*''); ^{13}C NMR (75 MHz, CD_3OD, 25°C): δ 171.9 (C), 164.8 (C), 164.5 (C), 151.3 (C), 148.0 (CH), 139.1 (C), 129.4 (CH), 128.9 (CH), 128.8 (CH), 106.1 (C), 89.5 (CH), 87.7 (CH), 74.2 (CH_2), 72.3 (CH), 70.4 (CH_2), 62.8 (CH_2), 54.2 (CH), 53.0 (CH_3), 41.9 (CH_2); HRMS (ESI): *m/z*: calcd for $C_{21}H_{24}O_9N_3$: 462.1512 $[M-H]^-$; found: 462.1518.

Experimental details of the DMTr-protected modified nucleoside (9): Reaction of product **8** (90 mg, 0.20 mmol) and DMTr-Cl (152 mg, 0.45 mmol) according to the general procedure described above, gave the deprotected nucleoside **9** as an off-white pink colored foam (122 mg, 82%). R_f (DCM: MeOH 9:1) = 0.57; ^{1}H NMR (300 MHz, $CDCl_3$, 25°C): δ 9.36 (d, *J* = 7.8 Hz, 1H, -N*H*), 8.56 (s, 1H, *H6*), 7.41-7.16 (band, 14H, -Ar*H*/-DMTr*H*), 6.83 (d, *J* = 8.5 Hz, 4H, -DMTr*H*), 6.15 (app t, *J* = 6.5 Hz, 1H, *H1*'), 4.84 (app dt, *J* = 3.7 and 7.8 Hz, 1H, *H7*), 4.56 (d AB, *J* = 12.2 Hz, 1H, *H9*'/*H9*''), 4.49 (d AB, *J* = 12.2 Hz, 1H, *H9*'/*H9*''), 4.32 (dt, *J* = 4.2 and 6.5 Hz, 1H, *H3*'), 3.95 (ddd, *J* = 4.2, 4.7 and 5.7 Hz, 1H, *H4*'), 3.92 (dd, *J* = 3.7 and 9.6 Hz, 1H, *H8*'/*H8*''), 3.78 (s, 3H, -OCH_3), 3.77 (s, 3H, -OCH_3), 3.73 (s, 3H, -OCH_3), 3.71 (dd, *J* = 3.7 and 9.6 Hz, 1H, *H8*'/*H8*''), 3.48 (dd AB, *J* = 4.7 and 10.4 Hz, 1H, *H5*'/*H5*''), 3.36 (dd AB, *J* = 5.7 and 10.4 Hz, 1H, *H5*'/*H5*''), 2.47 (ddd AB, *J* = 4.2, 6.5 and 14.0 Hz, 1H, *H2*'/*H2*''), 2.22 (dt AB, *J* = 6.5 and 14.0 Hz, 1H, *H2*'/*H2*''); ^{13}C NMR (75 MHz, $CDCl_3$, 25°C): δ 170.7 (C), 162.6 (C), 161.7 (C), 158.8 (C), 149.6 (C), 146.2 (CH), 144.7 (C), 137.8 (C), 135.7 (C), 130.2 (CH), 130.1 (CH), 128.5 (CH), 128.2 (CH), 128.1 (CH), 127.9 (CH), 127.8 (CH), 127.1 (CH), 113.5 (CH), 106.1 (C), 87.1 (C), 86.4 (CH), 85.8 (CH), 73.4 (CH_2), 72.7 (CH), 69.6 (CH_2), 63.8 (CH_2), 55.4 (CH_3), 53.0 (CH), 52.7 (CH_3), 40.6 (CH_2); HRMS (ESI): *m/z*: calcd for $C_{42}H_{42}O_{11}N_3$: 764.2819 $[M-H]^-$; found: 764.2814.

Synthesis of the lysine modified nucleoside (11): Reaction of 5-iodo-3',5'-(*O*-di-*tert*-butyldimethylsilyl)-2'-deoxyuridine (**1**, 722 mg, 1.2 mmol) and the commercialy available protected lysine **10** (1.04 g, 3.5 mmol) according to the general procedure described above, gave the deprotected nucleoside **11** as a yellow foam (864 mg, 90%). R_f (DCM: MeOH 95:5)

= 0.33; ^{1}H NMR (300 MHz, $CDCl_3$, 25°C): δ 9.86 (s br, 1H, pyrimidine-N*H*), 9.01 (d, *J* = 7.6 Hz, 1H, -N*H*), 8.59 (s, 1H, *H6*), 7.31-7.22 (band, 5H, -Ar*H*), 6.15 (dd, *J* = 5.7 and 7.3 Hz, 1H, *H1*'), 5.01 (s, 2H, *H12*'/*H12*''), 4.64 (dt, *J* = 5.3 and 7.6 Hz, 1H, *H7*), 4.34 (dt, *J* = 2.0 and 5.6 Hz, 1H, *H3*'), 3.98 (td, *J* = 2.0 and 3.6 Hz, 1H, *H4*'), 3.72 (d, *J* = 3.6 Hz, 2H, *H5*'/*H5*''), 3.67 (s, 3H, -OC*H3*), 3.14 (app q, *J* = 6.3 Hz, 2H, *H11*'/*H11*''), 2.34 (ddd AB, *J* = 2.0, 5.7 and 13.2 Hz, 1H, *H2*'/*H2*''), 1.99 (ddd AB, *J* = 5.5, 7.3 and 13.2 Hz, 1H, *H2*'/*H2*''), 1.94-1.70 (band, 2H, *H8*'/*H8*''), 1.48 (m, 2H, *H10*'/*H10*''), 1.36 (m, 2H, *H9*'/*H9*''), 0.84 (s, 9H, -*tBu*), 0.81 (s, 9H, -*tBu*), 0.03 (s br, 9H, -C*H3*), 0.02 (s, 3H, -C*H3*); ^{13}C NMR (75 MHz, $CDCl_3$, 25°C): δ 172.3 (C), 163.1 (C), 161.3 (C), 156.4 (C), 149.4 (C), 146.5 (CH), 136.6 (C), 128.4 (CH), 128.0 (CH), 105.6 (C), 88.8 (CH), 86.9 (CH), 72.8 (CH), 66.5 (CH_2), 63.1 (CH_2), 52.3 (CH_3), 52.0 (CH), 41.5 (CH_2), 40.7 (CH_2), 31.7 (CH_2), 29.3 (CH_2), 25.9 (CH_3), 25.7 (CH_3), 22.4 (CH_2), 18.3 (C), 17.9 (C), -4.8 (CH_3), -4.9 (CH_3), -5.5 (CH_3), -5.7 (CH_3); HRMS (ESI): *m/z*: calcd for $C_{37}H_{61}O_{10}N_4Si_2$: 777.3926 $[M+H]^+$; found: 777.3936.

Experimental details of the deprotected product (12): Reaction of product **11** (320 mg, 0.41 mmol) and $Et_3N·3HF$ (0.47 mL, 2.9 mmol) with an additional amount of dry Et_3N (0.40 mL, 3.0 mmol) according to the general procedure described above, gave the deprotected nucleoside **12** as a white foam (97 mg, 43%). R_f (DCM: MeOH 9:1) = 0.37; ^{1}H NMR (300 MHz, CD_3OD, 25°C): δ 8.84 (s, 1H, *H6*), 7.30 (m, 5H, -Ar*H*), 6.24 (app t, *J* = 6.4 Hz, 1H, *H1*'), 5.05 (s, 2H, *H12*'/*H12*''), 4.59 (dd, *J* = 5.3 and 7.7 Hz, 1H, *H7*), 4.39 (dt, *J* = 3.4 and 6.4 Hz, 1H, *H3*'), 3.99 (app q, *J* = 3.4 Hz, 1H, *H4*'), 3.81 (dd AB, *J* = 3.4 and 11.9 Hz, 1H, *H5*'/*H5*''), 3.73 (dd AB, *J* = 3.4 and 11.3 Hz, 1H, *H5*'/*H5*''), 3.73 (s, 3H, -OC*H3*), 3.11 (app t, *J* = 6.6 Hz, 2H, *H11*'/*H11*''), 2.38 (ddd AB, *J* = 3.4, 6.4 and 13.5 Hz, 1H, *H2*'/*H2*''), 2.23 (dt AB, *J* = 6.4 and 13.5 Hz, 1H, *H2*'/*H2*''), 1.97-1.75 (band, 2H, *H8*'/*H8*''), 1.52 (m, 2H, *H10*'/*H10*''), 1.41 (m, 2H, *H9*'/*H9*''); ^{13}C NMR (75 MHz, CD_3OD, 25°C): δ 173.8 (C), 164.8 (C), 164.4 (C), 158.9 (C), 151.2 (C), 148.0 (CH), 138.5 (C), 129.5 (CH), 129.0 (CH), 128.7 (CH), 106.0 (C), 89.5 (CH), 87.8 (CH), 72.3 (CH), 67.3 (CH_2), 62.8 (CH_2), 53.6 (CH), 52.8 (CH_3), 41.9 (CH_2), 41.4 (CH_2), 32.7 (CH_2), 30.4 (CH_2), 23.7 (CH_2); HRMS (ESI): *m/z*: calcd for $C_{25}H_{33}O_{10}N_4$: 549.2196 $[M+H]^+$; found: 549.2191.

Synthesis of the DMTr-protected lysine modified nucleoside (13): Reaction of the modified nucleoside **12** (88 mg, 0.16 mmol) and DMTr-Cl (143 mg, 0.42 mmol) according to the general procedure described above, gave the deprotected nucleoside **13** as a white foam (13 mg, 9%). R_f (DCM: MeOH 9:1) = 0.38; ^{1}H NMR (300 MHz, $CDCl_3$, 25°C): δ 9.09 (d, *J* =

7.4 Hz, 1H, -N*H*), 8.58 (s, 1H, *H6*), 7.40-7.15 (band, 14H, -DMTr*H*/ -Ar*H*), 6.82 (d, 4H, *J* = 8.9 Hz, -DMTr*H*), 6.15 (app t, *J* = 6.2 Hz, 1H, *H1*'), 5.06 (s br, 2H, *H12*'/*H12*''), 4.85 (app t, *J* = 5.3 Hz, 2H, *H11*'/*H11*''), 4.67 (td, *J* = 5.0 and 7.4 Hz, 1H, *H7*), 4.29 (dt, *J* = 4.5 and 5.5 Hz, 1H, *H3*'), 4.01 (q, *J* = 4.5 Hz, 1H, *H4*'), 3.77 (s, 6H, -OCH_3), 3.71 (s, 3H, -OCH_3), 3.43 (dd AB, *J* = 4.5 and 10.2 Hz, 1H, *H5*'/*H5*''), 3.36 (dd AB, *J* = 4.5 and 10.2 Hz, 1H, *H5*'/*H5*''), 3.08 (m, 2H, *H10*'/*H10*''), 2.50 (ddd AB, *J* = 4.5, 6.2 and 13.7 Hz, 1H, *H2*'/*H2*''), 2.19 (m, 1H, *H2*'/*H2*''), 1.87 (m, 1H, *H8*'/*H8*''), 1.76 (m, 1H, *H8*'/*H8*''), 1.35 (m, 2H, *H9*'/*H9*''); ^{13}C NMR (75 MHz, $CDCl_3$, 25°C): δ 172.6 (C), 163.2 (C), 158.7 (C), 149.7 (C), 144.8 (C), 135.8 (C), 130.2 (CH), 128.6 (CH), 128.2 (CH), 113.4 (CH), 105.9 (C), 87.0 (C), 86.5 (CH), 86.0 (CH), 72.6 (CH), 66.7 (CH_2), 63.9 (CH_2), 55.4 (CH_3), 53.2 (CH_2), 52.5 (CH/CH_3), 40.9 (CH_2), 32.1 (CH_2), 29.5 (C), 22.7 (CH_2); HRMS (ESI): *m/z*: calcd for $C_{46}H_{49}O_{12}N_4$: 849.3347 $[M-H]^-$; found: 849.3365.

Product 3: ^{1}H NMR, 300 MHz, $CDCl_3$, 25 °C

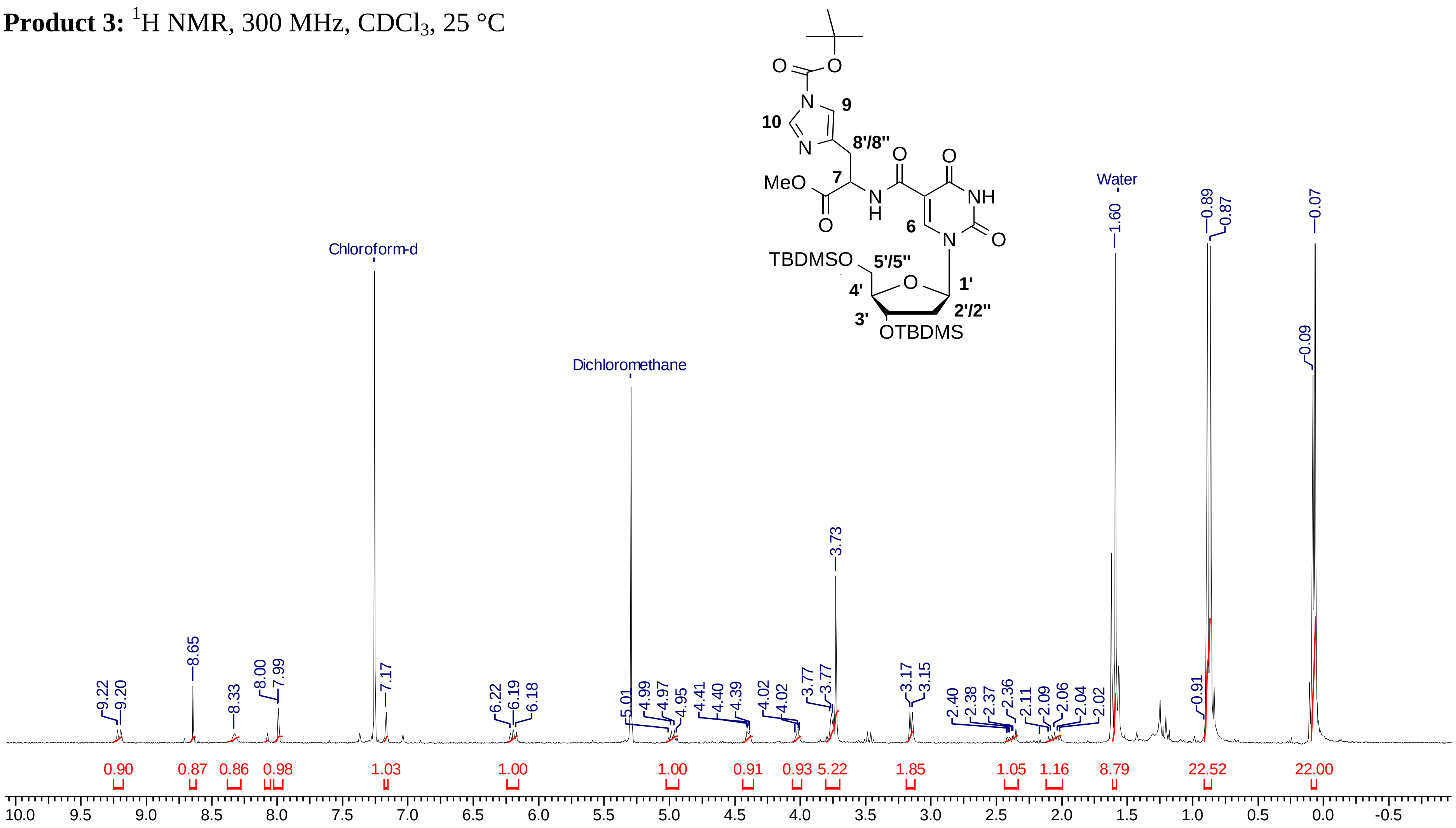

Product 3: ^{13}C NMR, 300 MHz, $CDCl_3$, 25 °C

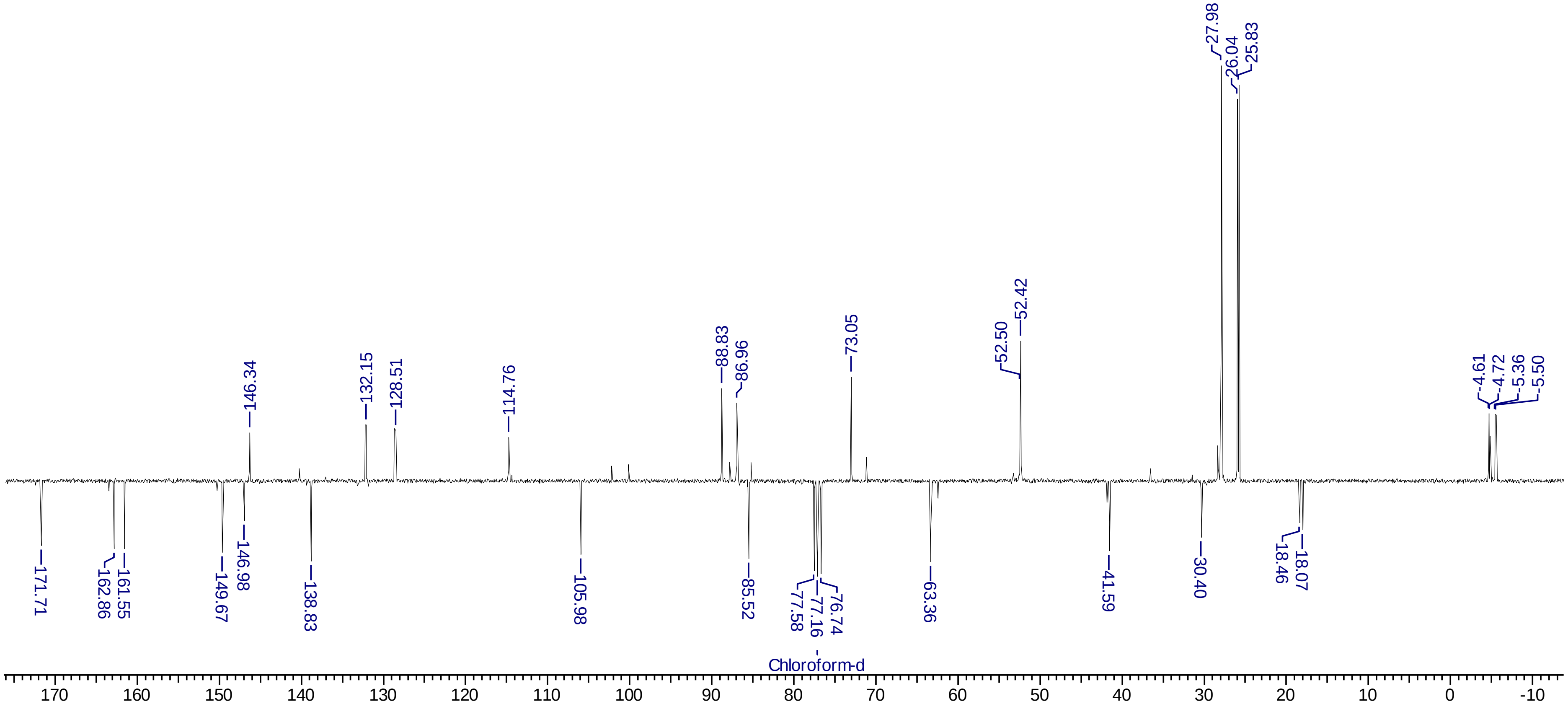

Product 4: ^{1}H NMR, 300 MHz, $CDCl_3$, 25 °C

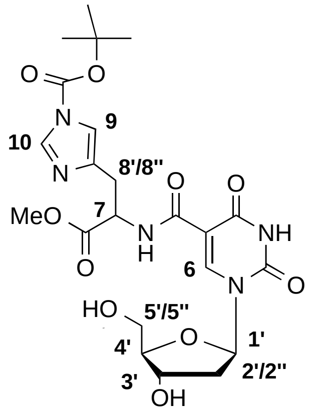

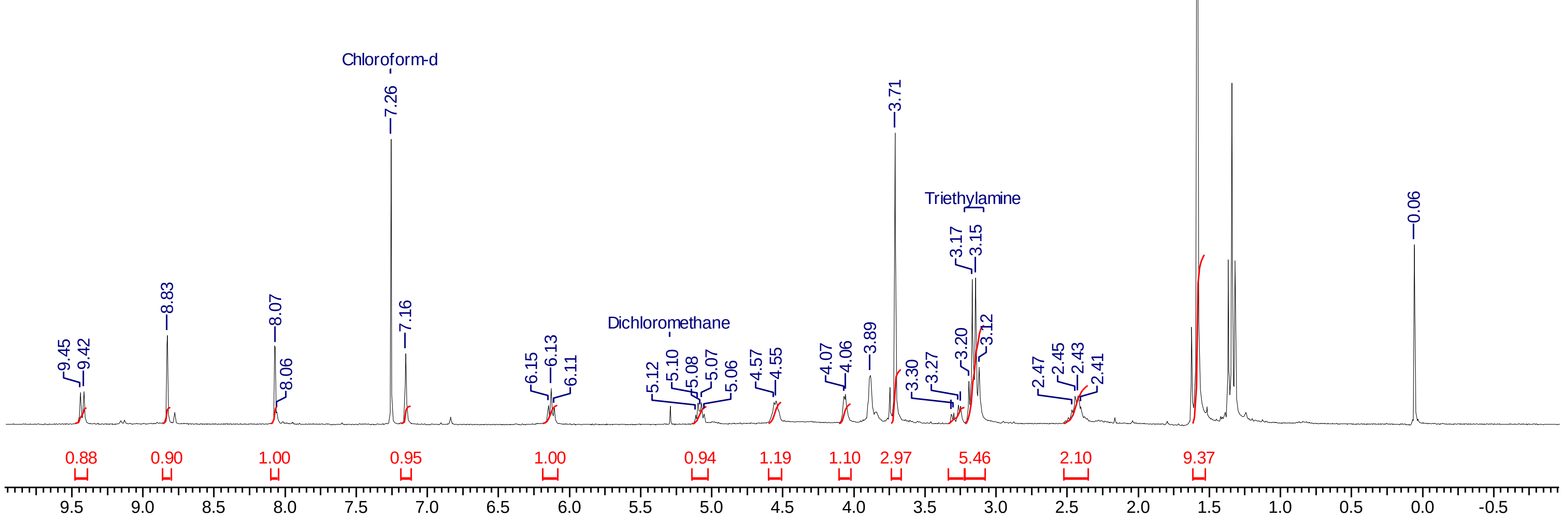

Product 4: ^{13}C NMR, 300 MHz, $CDCl_3$, 25 °C

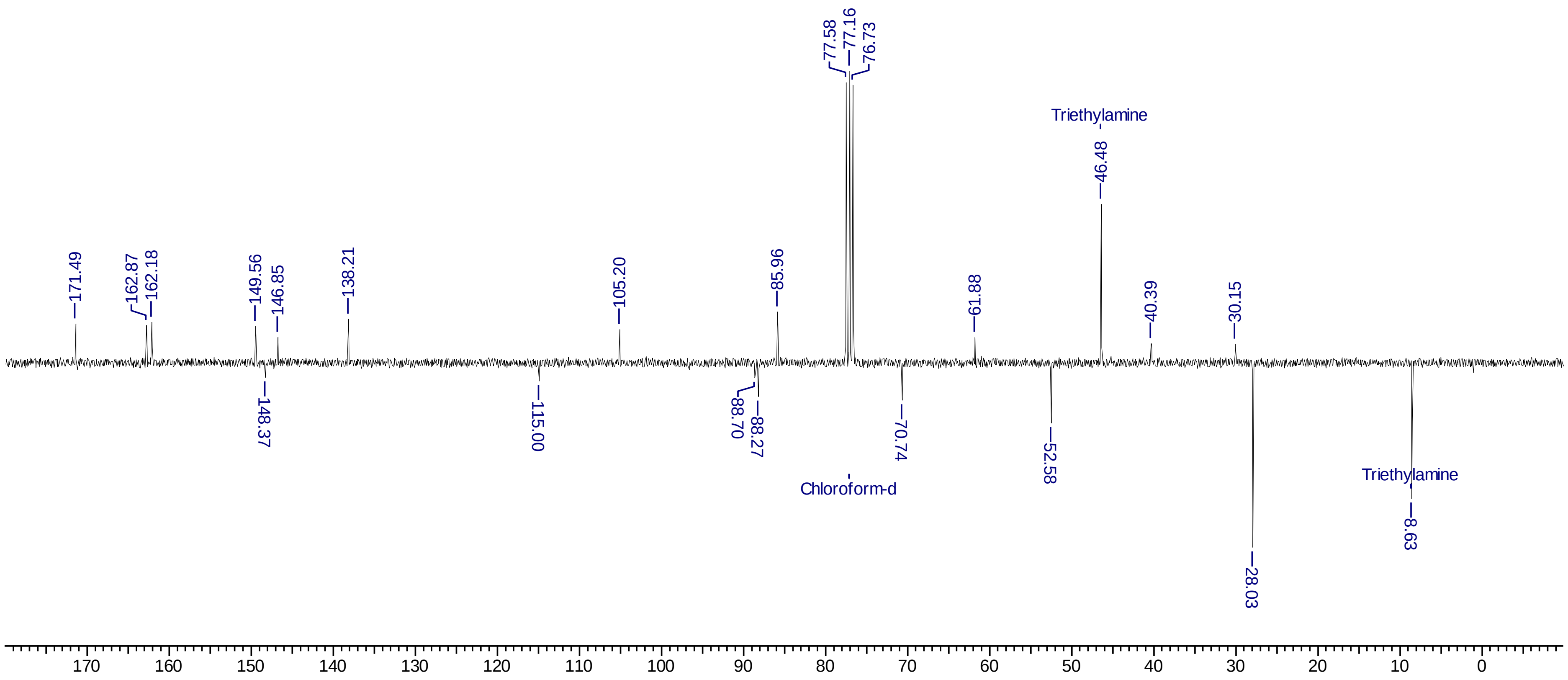

Product 5: ^{1}H NMR, 300 MHz, $CDCl_3$, 25 °C

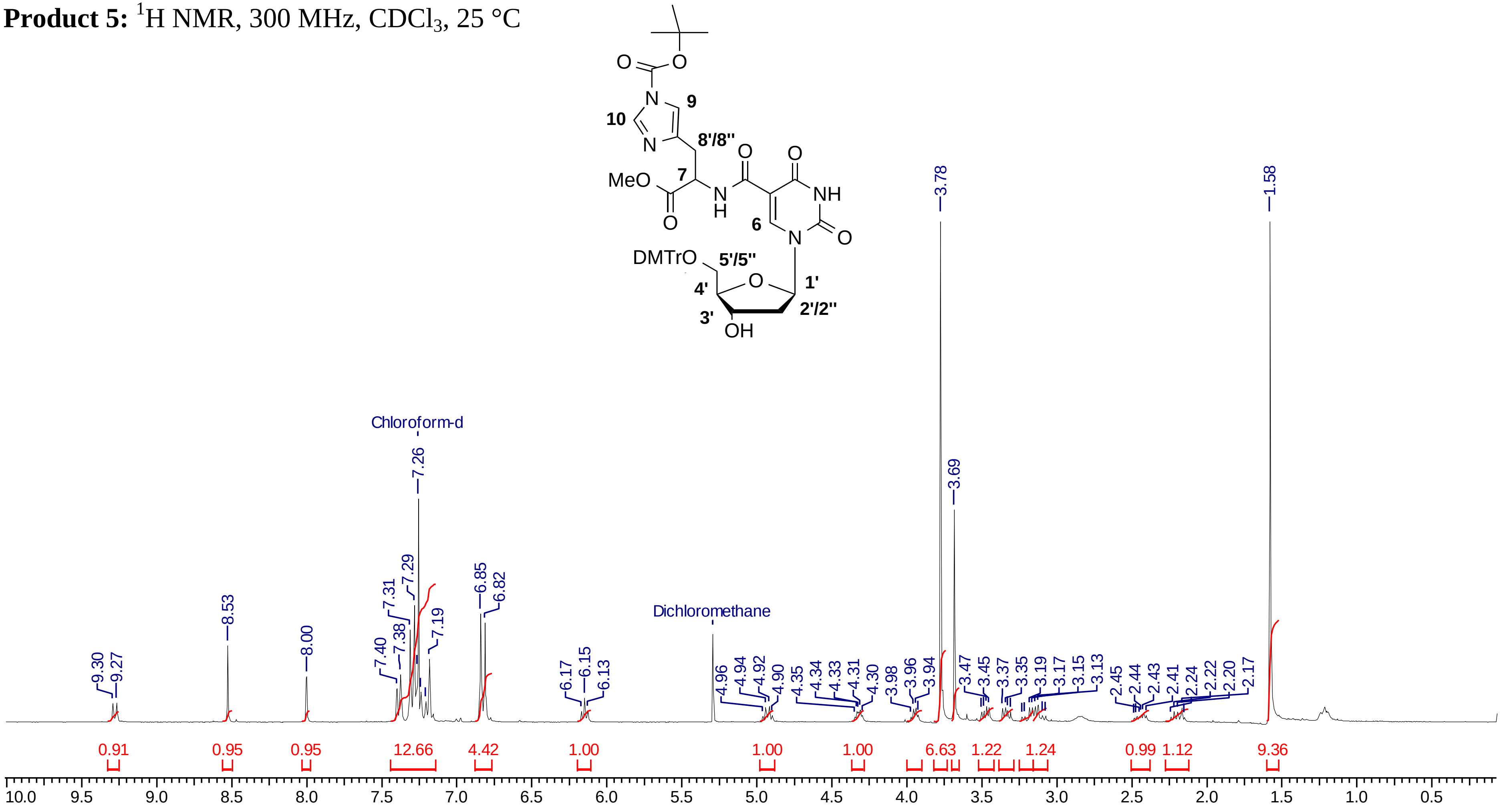

Product 5: ^{13}C NMR, 300 MHz, $CDCl_3$, 25 °C

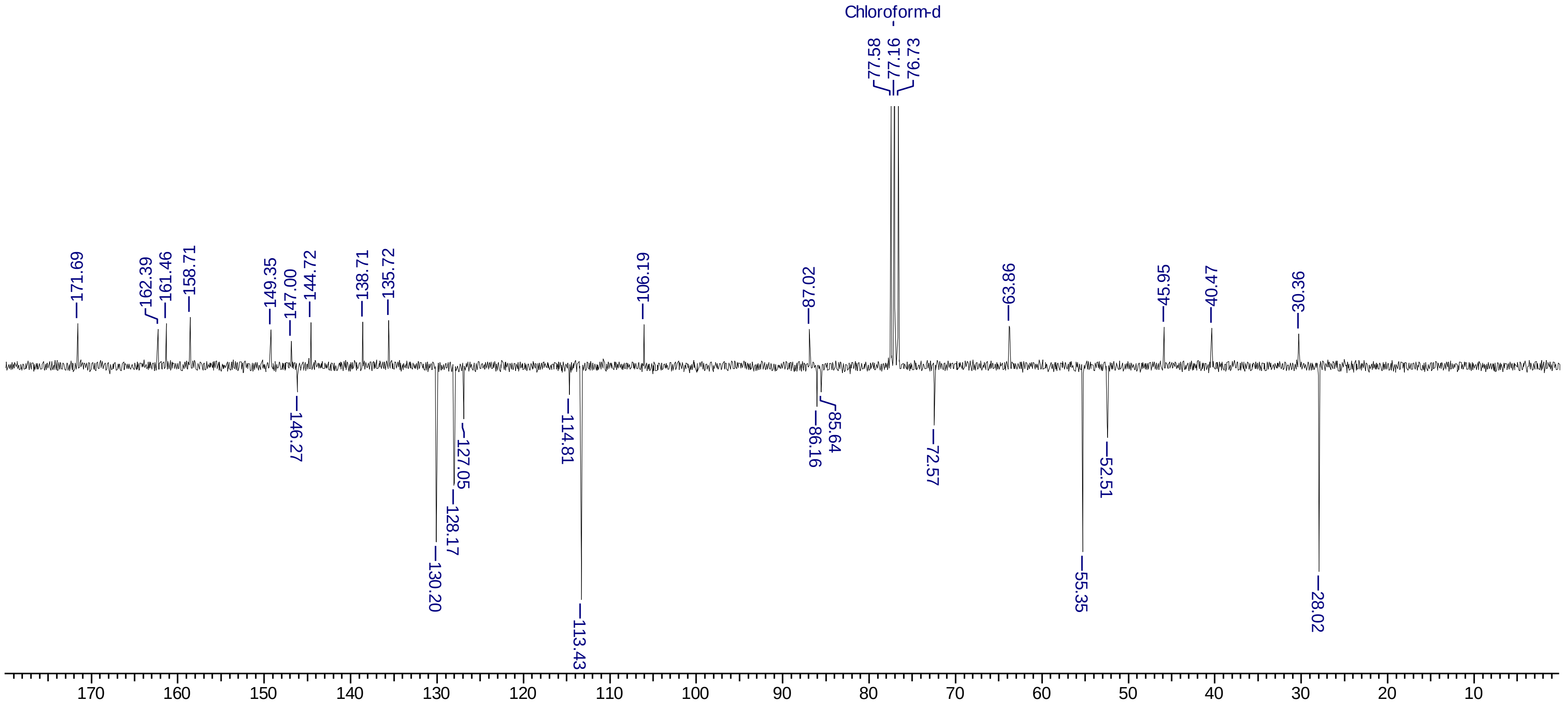

Product 7: ^{1}H NMR, 300 MHz, $CDCl_3$, 25 °C

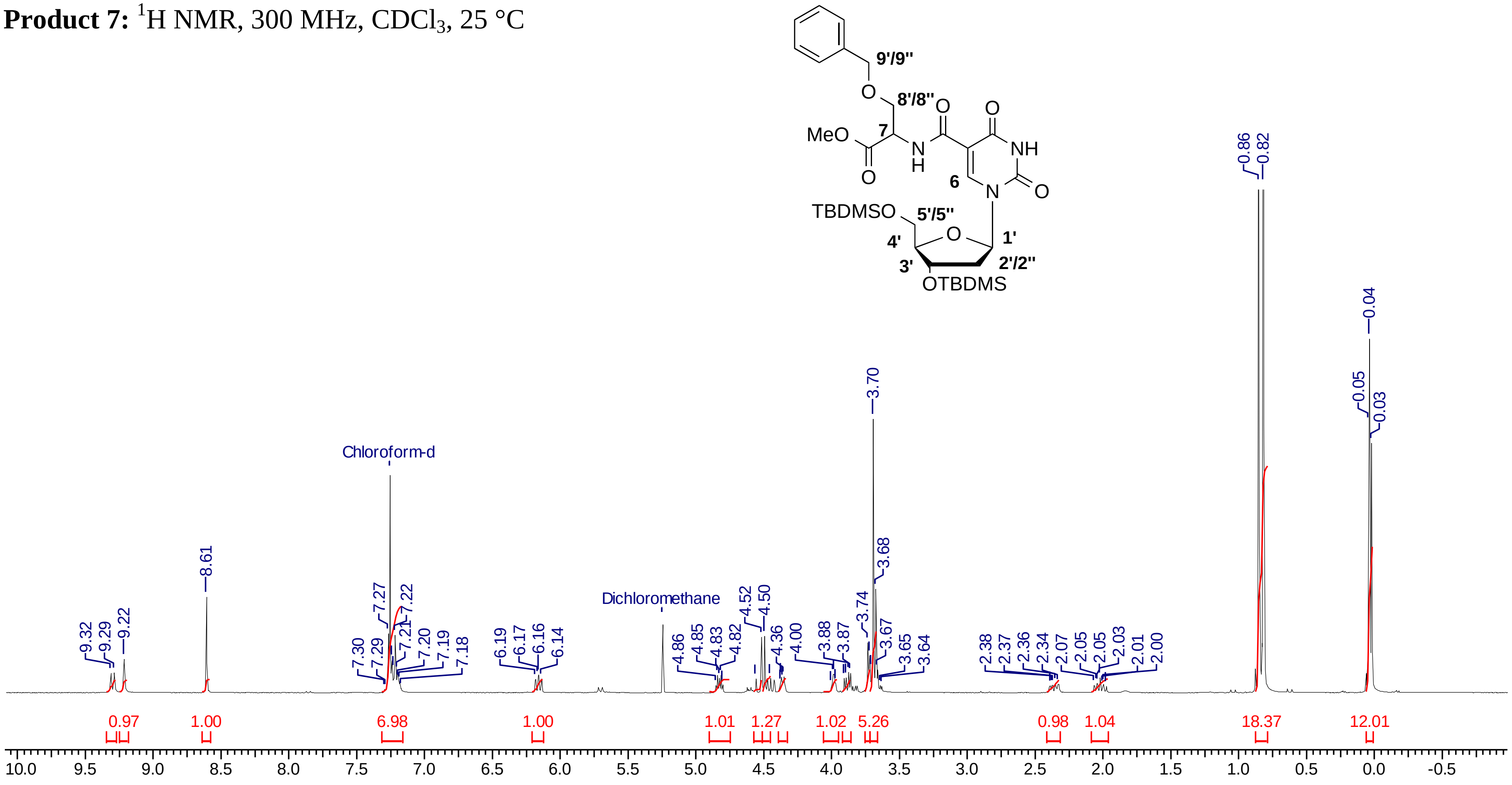

Product 7: ^{13}C NMR, 300 MHz, $CDCl_3$, 25 °C

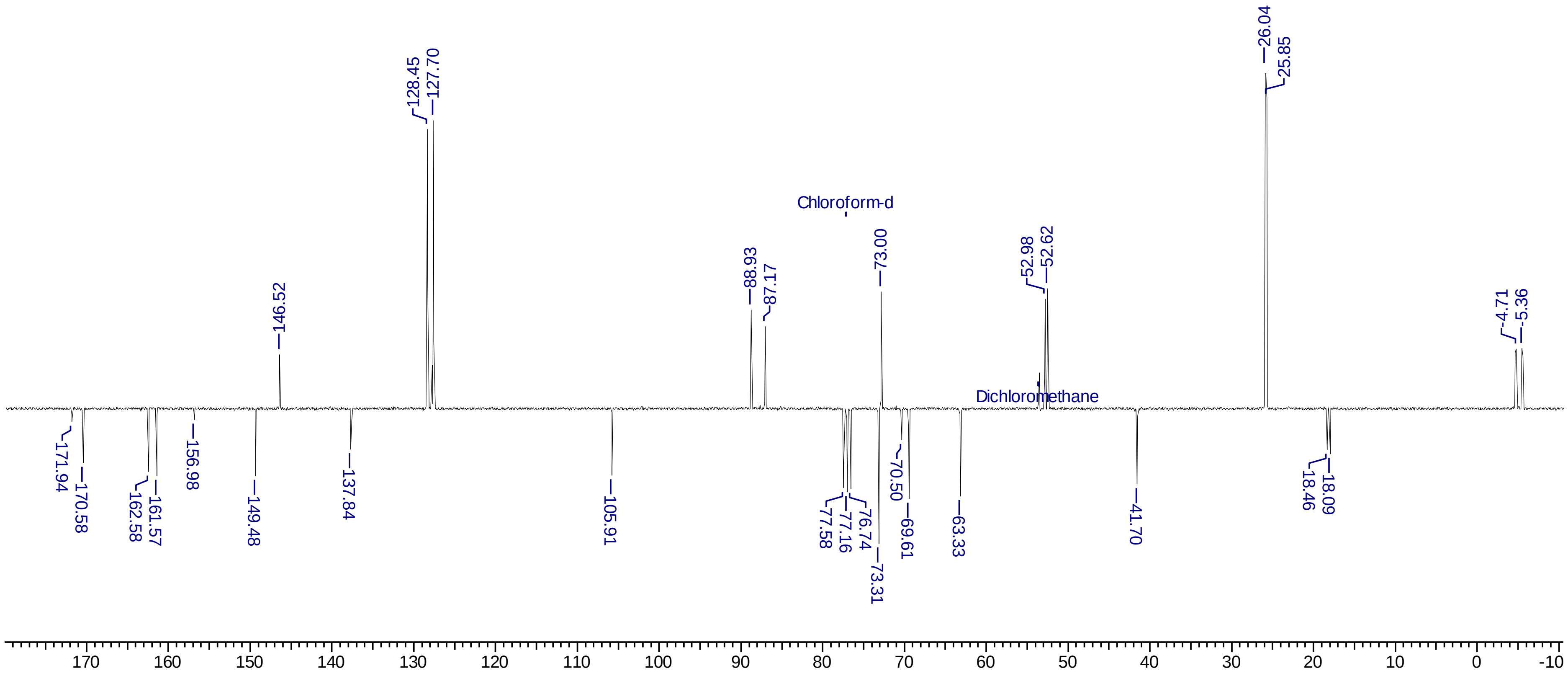

Product 8: ^{1}H NMR, 300 MHz, CD_3OD, 25 °C

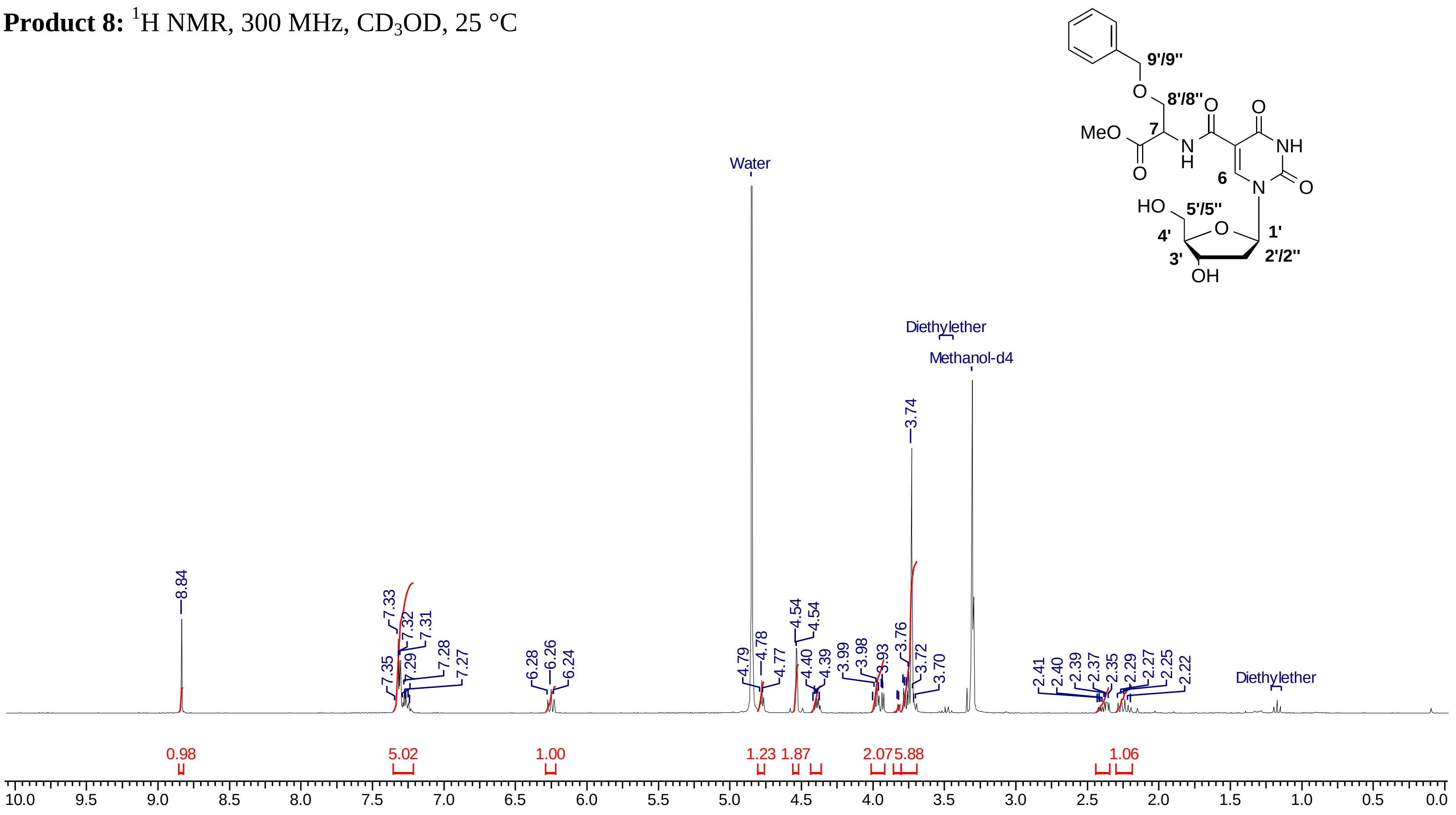

Product 8: ^{13}C NMR, 300 MHz, CD_3OD, 25 °C

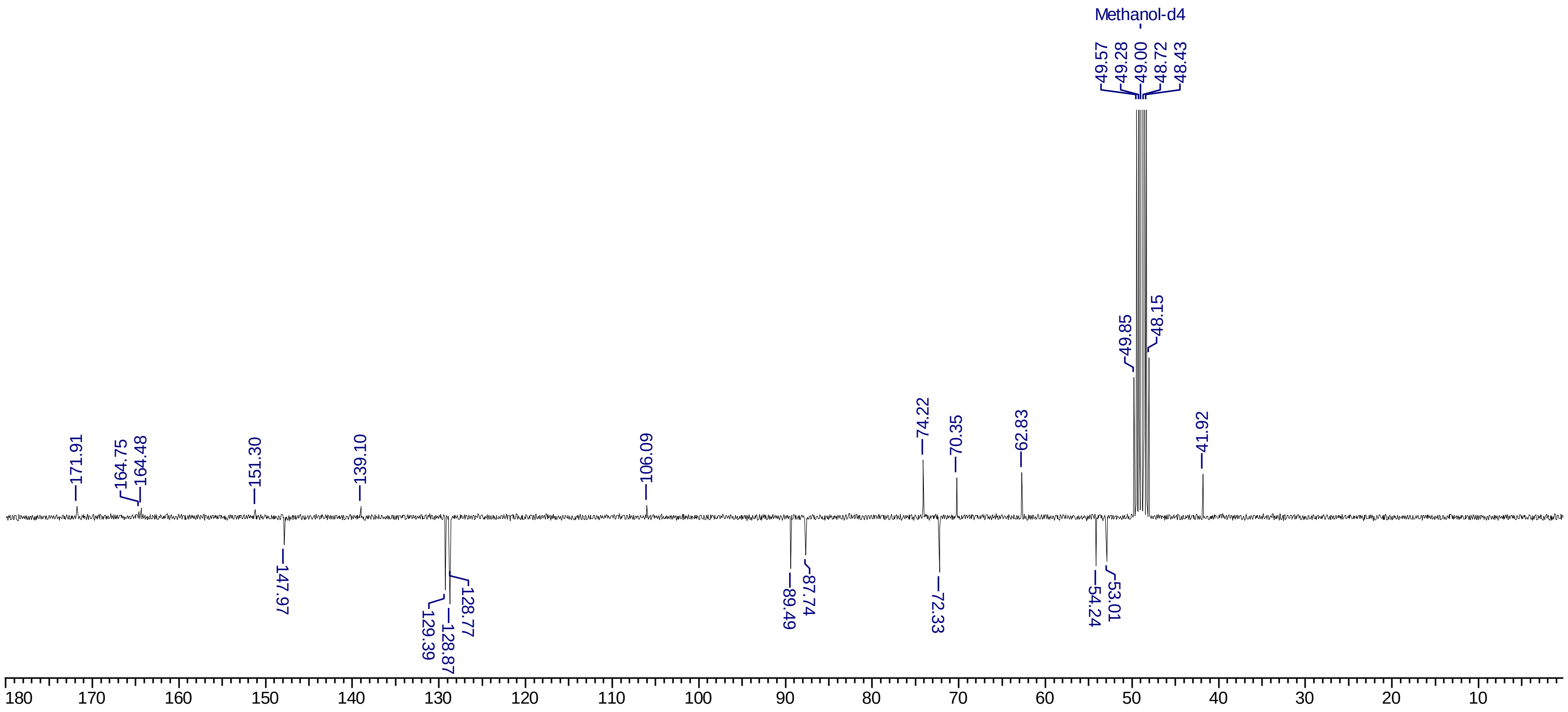

Product 9: ^{1}H NMR, 300 MHz, $CDCl_3$, 25 °C

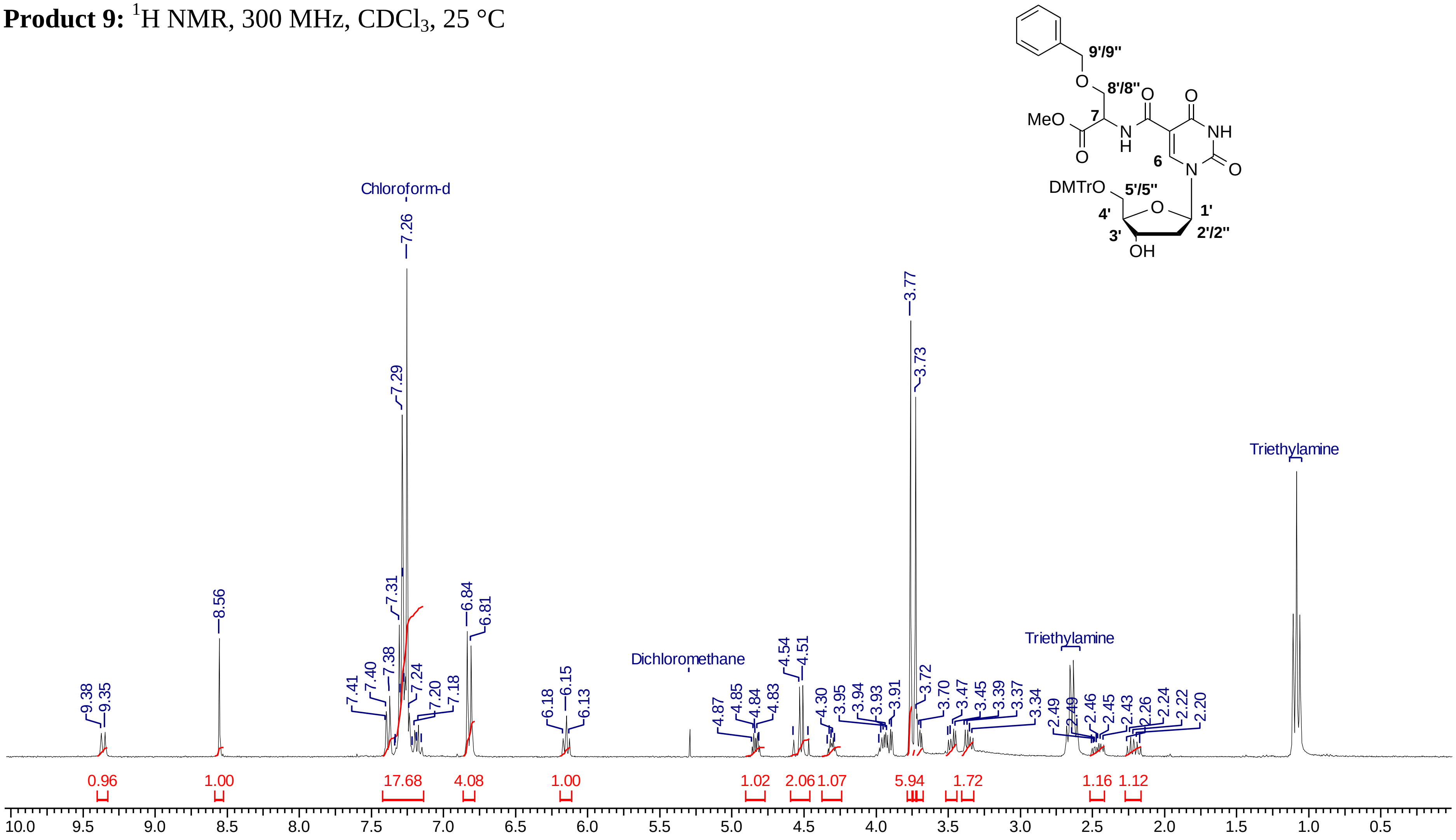

Product 9: ^{13}C NMR, 300 MHz, $CDCl_3$, 25 °C

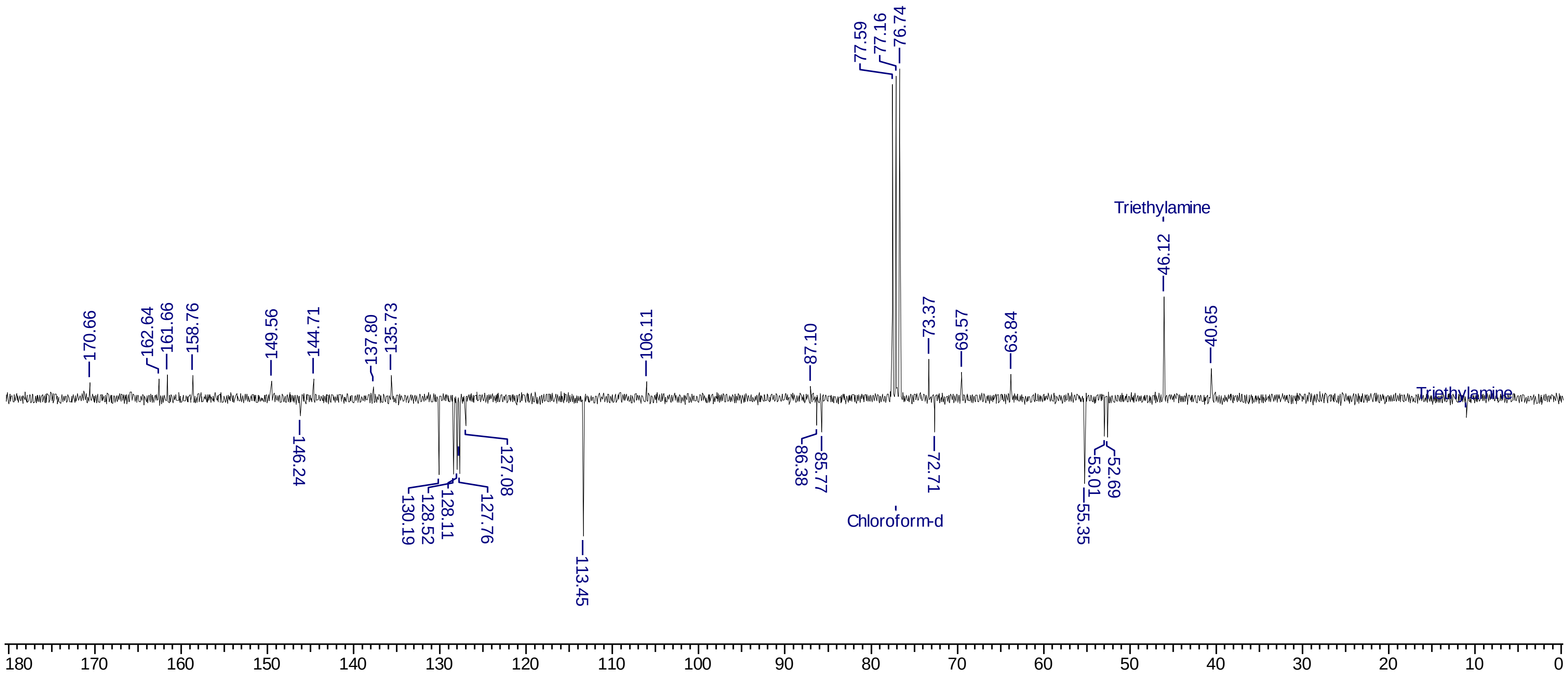

Product 11: ^{1}H NMR, 300 MHz, $CDCl_3$, 25 °C

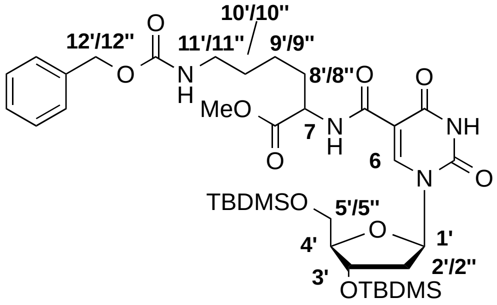

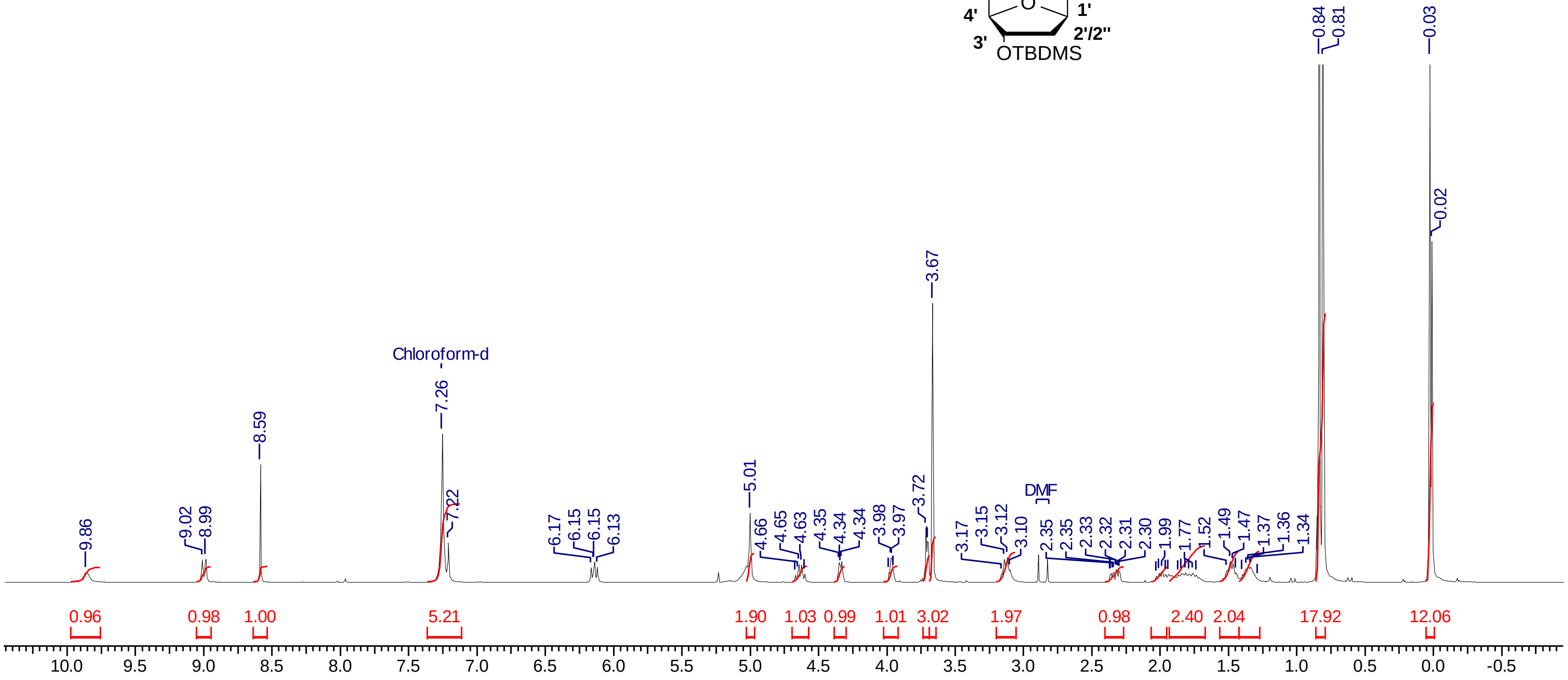

Product 11: ^{13}C NMR, 300 MHz, $CDCl_3$, 25 °C

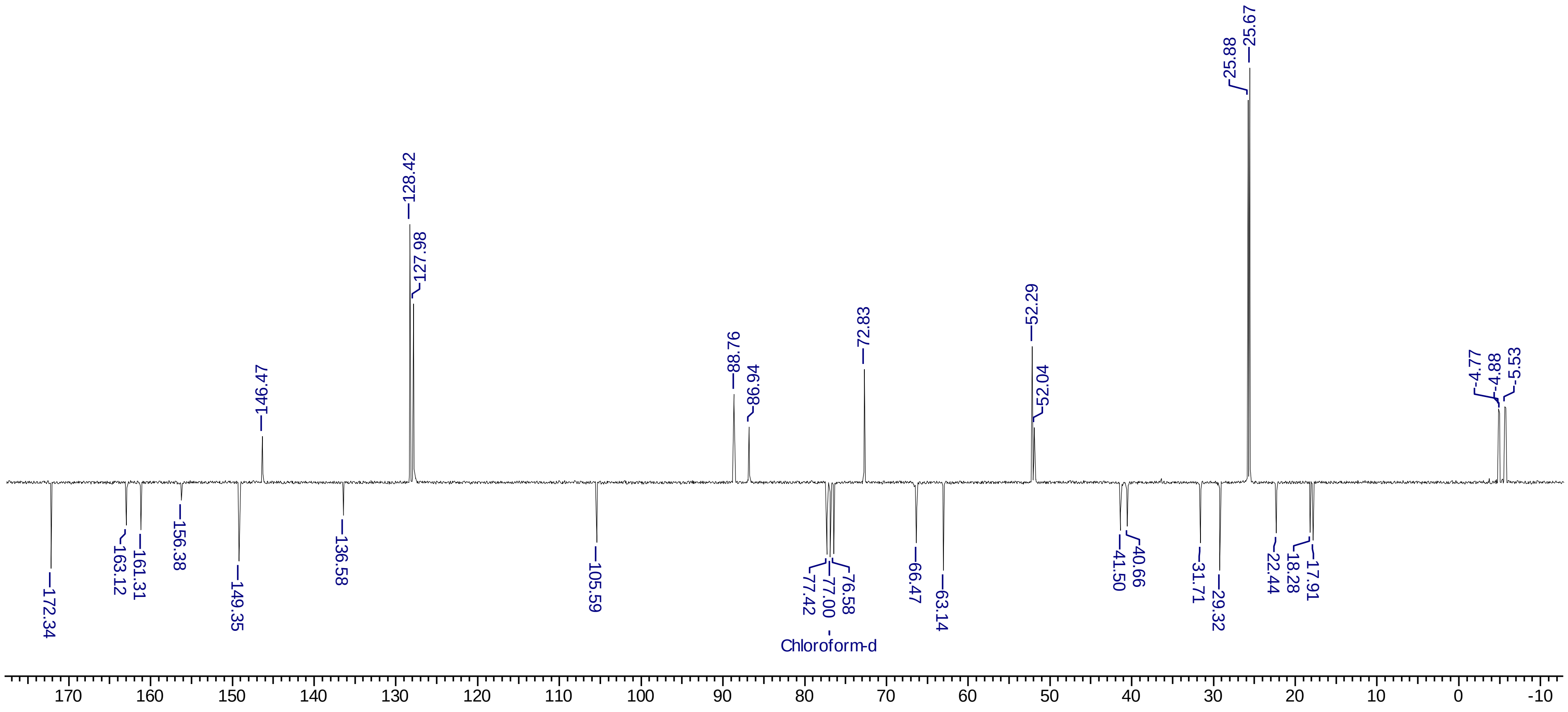

Product 12: ^{1}H NMR, 300 MHz, CD_3OD, 25 °C

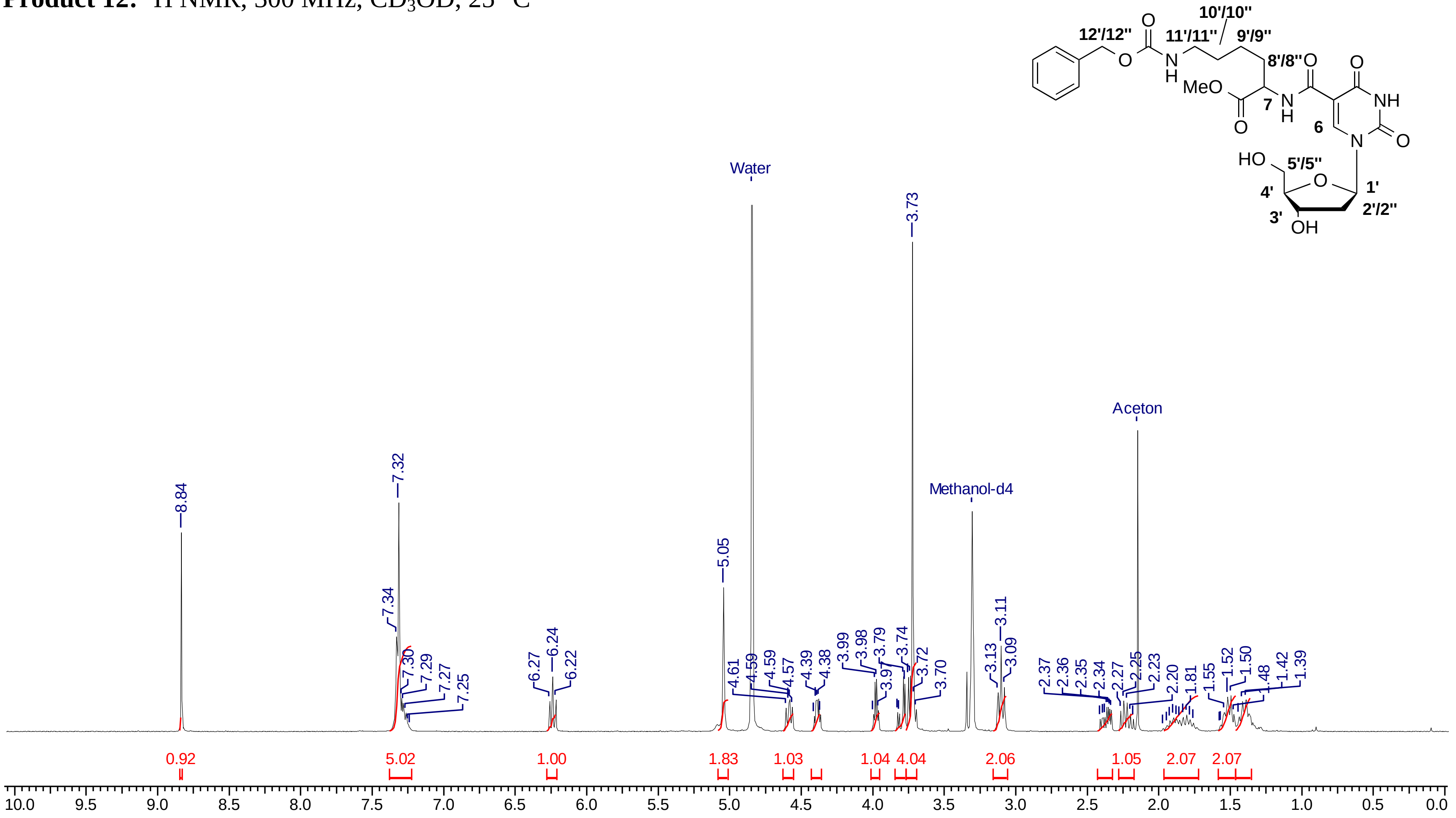

Product 12: ^{13}C NMR, 300 MHz, CD_3OD, 25 °C

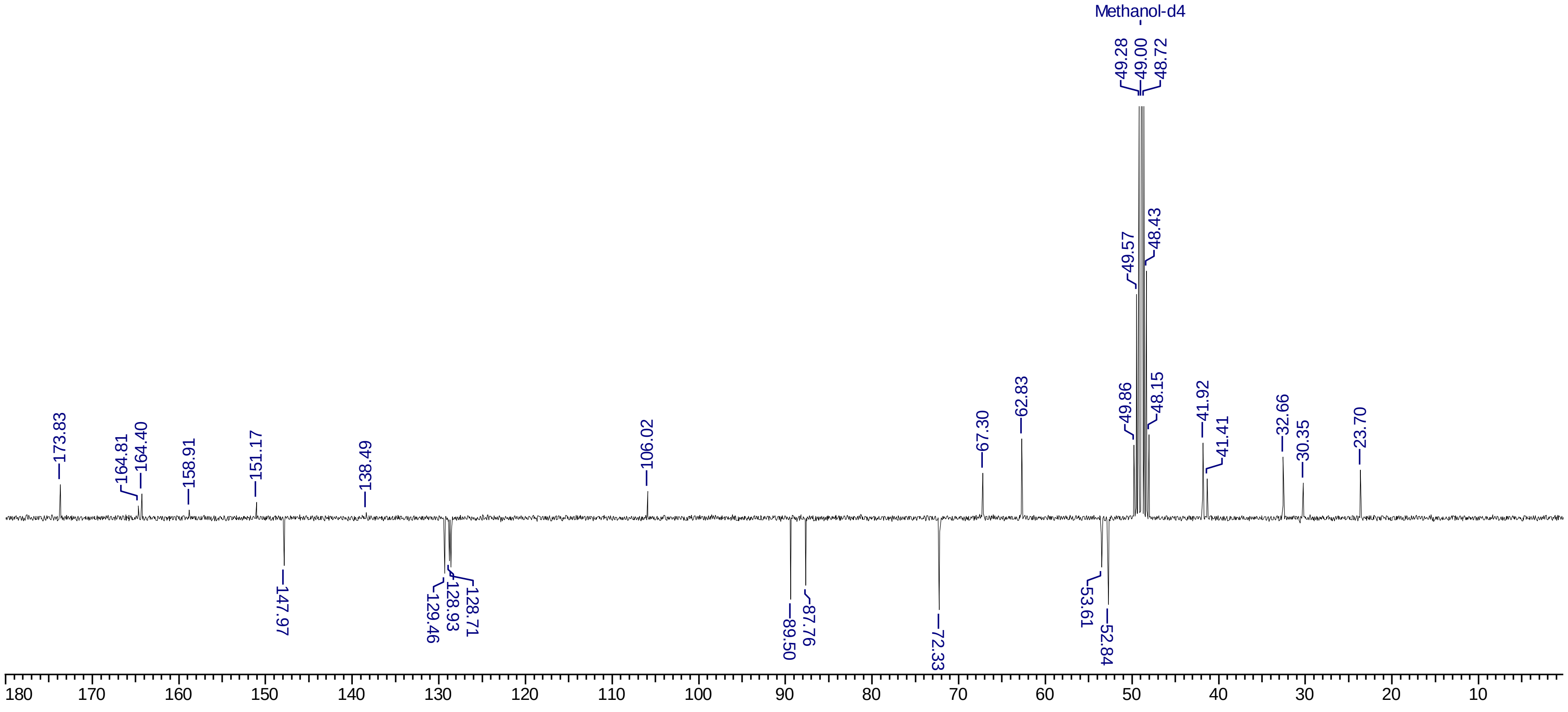

Product 13: ^{1}H NMR, 300 MHz, $CDCl_3$, 25 °C

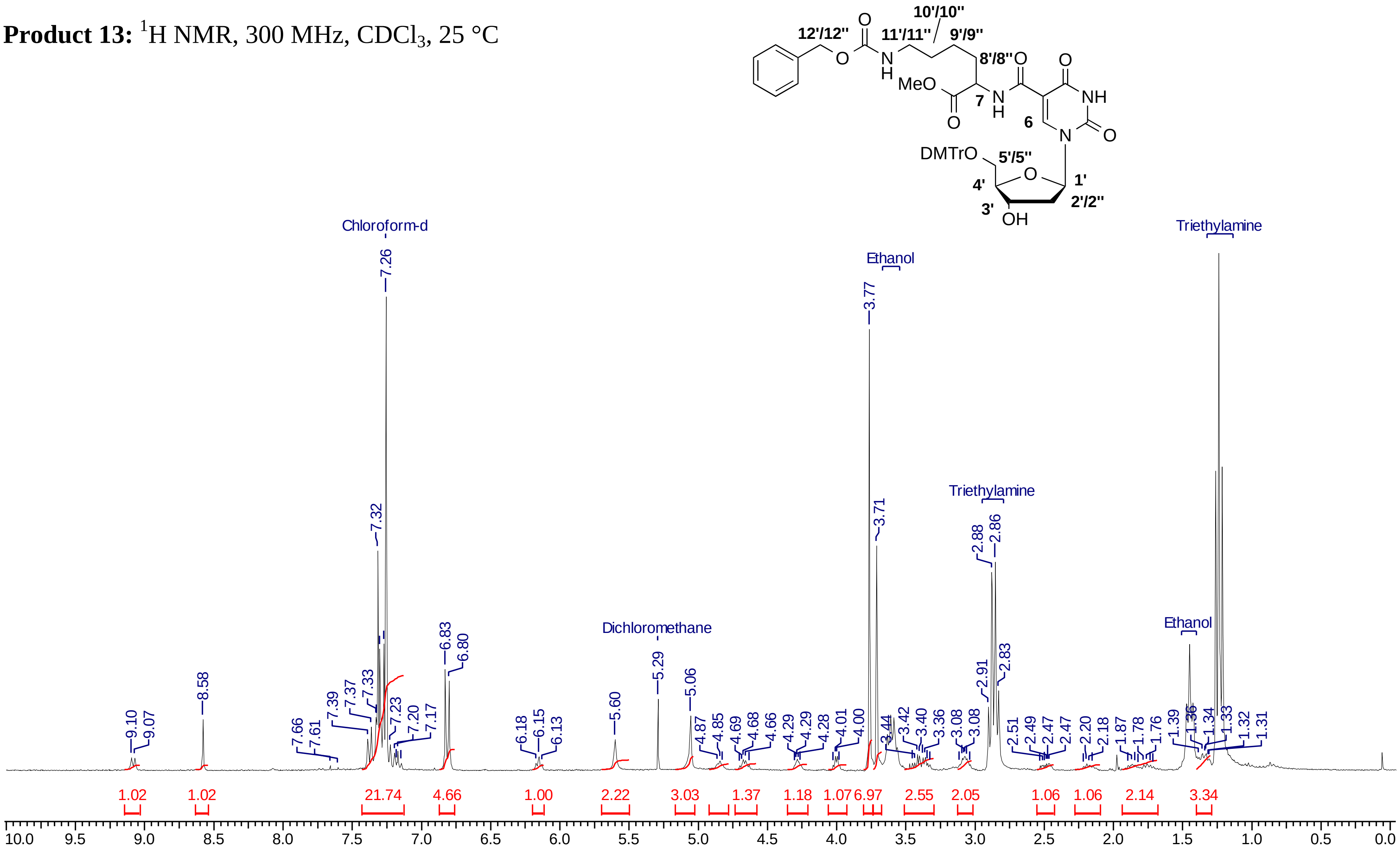

Product 13: ^{13}C NMR, 300 MHz, $CDCl_3$, 25 °C

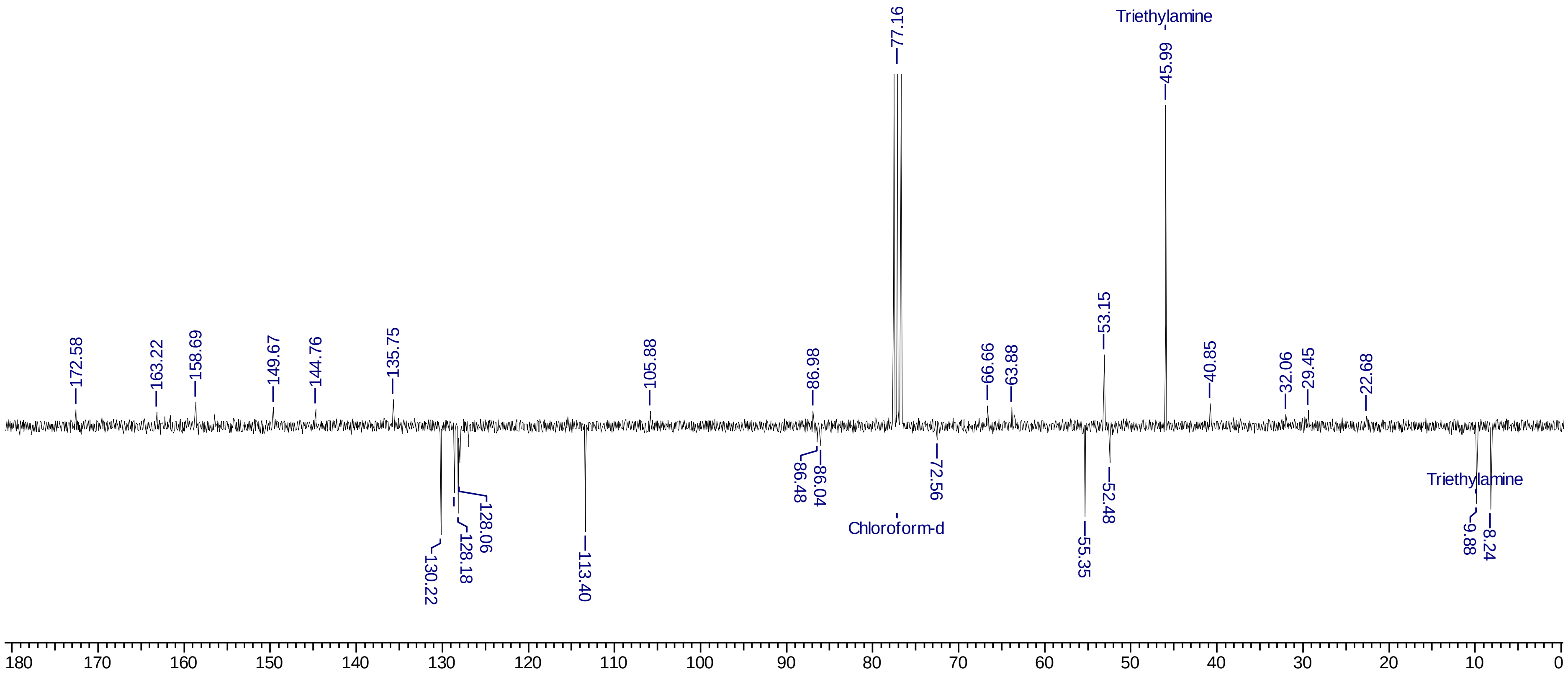

Supplement: File 1 — Experimental procedures, characterization data, and 1H and 13C NMR spectra of new compounds. [file Beilstein_J_Org_Chem-10-2566-s001.pdf]
